# Saddle Slow Manifolds and Canard Orbits in $\mathbb{R}^{4}$ and Application to the Full Hodgkin–Huxley Model

**DOI:** 10.1186/s13408-018-0060-1

**Published:** 2018-04-19

**Authors:** Cris R. Hasan, Bernd Krauskopf, Hinke M. Osinga

**Affiliations:** Department of Mathematics, The University of Auckland, Auckland, New Zealand

**Keywords:** Slow–fast systems, Saddle slow manifolds, Canard orbits, Hodgkin–Huxley model, Mixed-mode oscillations

## Abstract

Many physiological phenomena have the property that some variables evolve much faster than others. For example, neuron models typically involve observable differences in time scales. The Hodgkin–Huxley model is well known for explaining the ionic mechanism that generates the action potential in the squid giant axon. Rubin and Wechselberger (Biol. Cybern. 97:5–32, 2007) nondimensionalized this model and obtained a singularly perturbed system with two fast, two slow variables, and an explicit time-scale ratio *ε*. The dynamics of this system are complex and feature periodic orbits with a series of action potentials separated by small-amplitude oscillations (SAOs); also referred to as mixed-mode oscillations (MMOs). The slow dynamics of this system are organized by two-dimensional locally invariant manifolds called slow manifolds which can be either attracting or of saddle type.

In this paper, we introduce a general approach for computing two-dimensional saddle slow manifolds and their stable and unstable fast manifolds. We also develop a technique for detecting and continuing associated canard orbits, which arise from the interaction between attracting and saddle slow manifolds, and provide a mechanism for the organization of SAOs in $\mathbb{R}^{4}$. We first test our approach with an extended four-dimensional normal form of a folded node. Our results demonstrate that our computations give reliable approximations of slow manifolds and canard orbits of this model. Our computational approach is then utilized to investigate the role of saddle slow manifolds and associated canard orbits of the full Hodgkin–Huxley model in organizing MMOs and determining the firing rates of action potentials. For *ε* sufficiently large, canard orbits are arranged in pairs of twin canard orbits with the same number of SAOs. We illustrate how twin canard orbits partition the attracting slow manifold into a number of ribbons that play the role of sectors of rotations. The upshot is that we are able to unravel the geometry of slow manifolds and associated canard orbits without the need to reduce the model.

## Introduction

In a wide variety of models, including of semiconductor lasers [2, 3], chemical reactions [4–6] and neurons [7–9], one finds large differences in time scales. For example, in neuron models, the action potential changes on a much faster time scale than ionic gating variables. This yields various types of complex oscillatory behavior such as spiking, bursting and mixed-mode oscillations (MMOs),which alternate between small-amplitude oscillations (SAOs) and large-amplitude oscillations (LAOs) [10]. Those phenomena can be modeled by a special class of dynamical systems called *slow–fast systems*, which have a group of fast and a group of slow variables, with a time-scale separation parameter *ε*. A common approach for studying slow–fast systems is geometric singular perturbation theory (GSPT) [11, 12] which exploits the time-scale differences and approximates a slow–fast system by a composition of lower-dimensional slow and fast subsystems that are easier to analyze.

In the simplest case, slow–fast systems feature one fast and one slow variables. One famous example is the FitzHugh–Nagumo model [13, 14], which is a simplified two-dimensional dynamical system that describes the evolution of the voltage potential and a single coupled slow gating variable. The voltage nullcline corresponds to the so-called *critical manifold*. The cubic shape of the critical manifold provides a mechanism for spike generation and *relaxation oscillations*. The notion relaxation oscillation was first introduced for the Van der Pol oscillator [15] to describe periodic orbits with slow and fast segments. Parameter variations may lead to transitions between an excitable rest state, small periodic orbits and large relaxation oscillations. The transformation of small periodic orbits of the FitzHugh–Nagumo model into large relaxation oscillations occurs within an exponentially small parameter range. In this parameter range, periodic orbits known as *canard cycles* follow the middle (repelling) branch of the critical manifold for a considerable amount of time. This phenomenon is called “canard explosion” and was first discovered by Benoît in 1981 [16]. The analysis of canards has since been prominent for understanding slow–fast systems.

Neuron models with one slow and two fast variables may feature high-frequency spikes separated by quiescent periods. This behavior is called “bursting”. Rinzel [17] introduced a scheme for classifying bursting mechanisms based on the bifurcation structure of the so-called fast subsystem where the slow variable is treated as a parameter. Based on this structure, Izhikevich [18] attempted to classify all such bursting patterns. The critical manifold in bursting models with one slow and two fast variables is a one-dimensional manifold of equilibrium points of the fast subsystem, and it often takes a cubic shape. In this setting, canard orbits feature segments that stay close to the middle (saddle) branch of the critical manifold for a significant amount of time. Canard orbits play a key role in organizing bursting patterns; namely, they allow for an abrupt increase of amplitude in a very small parameter range. This creates a mechanism for spike adding [19] in bursting models [9, 20–26]. A different mechanism specifically for the transition between spiking and bursting has been related to so-called torus canards, which are orbit segments that connect the stable and unstable branches of periodic orbits of the fast subsystem [27–31].

Systems with one fast and two slow variables may generate a robust mechanism for MMOs. The critical manifolds of such systems are typically two-dimensional folded surfaces. Attracting and repelling sheets of a folded critical manifold meet at fold curves. According to well-established results from Fenichel [11, 12], normally hyperbolic sheets of the critical manifold persist as two-dimensional slow manifolds, provided *ε* is sufficiently small. Intersections of attracting and repelling slow manifolds in this setting correspond to canard orbits. They provide a mechanism for SAOs in that canard orbits form boundaries between regions of different numbers of SAOs [32–34]. Global return mechanisms and canard-induced SAOs together generate MMOs for a wide range of mathematical and applied three-dimensional models [1, 9, 20, 35–44]. In support of theoretical work, numerical methods were developed to reveal the geometry of two-dimensional attracting and repelling slow manifolds, as well as canard orbits. These methods were employed to investigate numerous three-dimensional examples [35, 36, 41, 43, 45–48]. For a more detailed survey of MMOs in slow–fast systems see [10].

Mechanisms of bursting and MMOs in three-dimensional vector fields are relatively well understood. Nevertheless, there are many three-dimensional slow–fast systems [1, 37, 49, 50] that are actually reductions of higher-dimensional models. For example, the Hodgkin–Huxley model is a four-dimensional vector field that was originally formed to explain the ionic mechanism that generates the action potential in the squid giant axon. Rubin and Wechselberger [1] reduced this model to a three-dimensional vector field in order to understand the role of slow manifolds and canard orbits in organizing the dynamics of the model. In certain situations, the reduced models accurately capture the qualitative behavior of the original higher-dimensional models. However, important aspects of the dynamics may be lost after a reduction [51, 52]. Thus, it is very useful to be able to investigate a given system without applying reduction techniques first.

As a natural step for studying higher-dimensional models, we focus on systems with two fast and two slow variables. In particular, we aim to compute and visualize slow manifolds and associated canard orbits in four-dimensional vector fields without the need for any reduction. To this end, we introduce a method for computing two-dimensional saddle slow manifolds in $\mathbb{R}^{4}$. Computation of saddle slow manifolds is more challenging than that of attracting and repelling slow manifolds due to the existence of both contracting and expanding fast normal directions. This challenge can be overcome with a two-point boundary-value problem (2PBVP) setup. Namely, we compute two-dimensional submanifolds of saddle slow manifolds and their stable and unstable manifolds as families of orbits segments. An important difference with previous studies, is that, in systems with two fast and two slow variables, canard orbits are no longer intersections of slow manifolds. In this paper, we develop and present a homotopy method for computing canard orbits as orbits segments on the attracting slow manifold that stay close to the saddle slow manifolds for a significant amount of time.

We study two different four-dimensional vector fields that feature two-dimensional saddle slow manifolds. The first is an extended version of the normal form of a folded node [34, 47], where a fast variable with a stable fast direction is added. In this system, we implement and test our 2PBVP-based approach for computing saddle slow manifolds, and their stable and unstable fast manifolds, as well as associated canard orbits. We show that our methods give reliable approximations of slow manifolds and canard orbits. As a second example, we consider the four-dimensional Hodgkin–Huxley model [8]. Its dynamics is very complex and features periodic MMOs with a series of action potentials separated by subthreshold oscillations. Rubin and Wechselberger [42] nondimensionalized the Hodgkin–Huxley model and obtained a system with two fast and two slow variables, and a simple explicit time-scale separation parameter *ε*. However, they then focused on a further reduced three-dimensional version of this model. Here, we compute attracting and saddle slow manifolds, as well as associated canard orbits directly in the nondimensionalized four-dimensional Hodgkin–Huxley model. Indeed, we demonstrate that such computations can be done without the need to reduce the system first. Our results confirm and illustrate how slow manifolds and their associated canard orbits play a central role in organizing MMOs also in the four-dimensional model.

The outline of the paper is as follows. Section 2 reviews ideas and concepts from GSPT for systems with two fast and two slow variables. Section 3 begins with a 2PBVP setup for computing orbit segments in four-dimensional vector fields. This is followed by a general method for computing two-dimensional saddle slow manifolds, as well as subsets of their three-dimensional stable and unstable manifolds in systems with two fast and two slow variables. Our approach is then demonstrated and implemented for an extended four-dimensional normal form of a folded node. In Sect. 4, we examine the interaction between attracting and saddle slow manifolds. We also introduce a general approach for computing canard orbits in $\mathbb{R}^{4}$ and then test it for the extended normal form of a folded node. In Sect. 5, we investigate the underlying dynamics of the full four-dimensional Hodgkin–Huxley model. A bifurcation analysis of MMOs and a short review of GSPT for this model are presented first. Thereafter, we compute attracting and saddle slow manifolds and study their interactions. For a relatively large *ε*, we illustrate the geometry of so-called ribbons and their bounding twin canard orbits, as well as their role in organizing MMOs. Finally, we perform a continuation analysis of canard orbits of the model. Conclusions, a summary of results and directions for future work can be found in Sect. 6.

## Background on Geometric Singular Perturbation Theory

In this paper, we focus on four-dimensional slow–fast (singularly perturbed) systems that can be written in the form

1 $$ \textstyle\begin{cases} \dot{\mathbf{x}} = f(\mathbf{x},\mathbf{y},\lambda ), \\ \dot{\mathbf{y}} = \varepsilon g(\mathbf{x},\mathbf{y},\lambda ), \end{cases} $$

with fast variables $\mathbf{x} \in \mathbb{R}^{2}$, slow variables $\mathbf{y} \in \mathbb{R}^{2}$, parameter vector $\lambda \in \mathbb{R}^{p}$, a small parameter $0 < \varepsilon \ll 1$ representing the time-scale ratio, and sufficiently smooth functions $f : \mathbb{R}^{2} \times \mathbb{R}^{2} \times \mathbb{R}^{p} \mapsto \mathbb{R}^{2}$ and $g : \mathbb{R}^{2} \times \mathbb{R}^{2} \times \mathbb{R}^{p} \mapsto \mathbb{R}^{2}$. The overdot denotes the derivative with respect to the fast time scale *t*.

Geometric singular perturbation theory (GSPT) exploits the separation of different time scales in order to explain the complex dynamics of slow–fast systems. The idea behind GSPT is to analyze two lower-dimensional subsystems of the singular limit and gather information from both subsystems to understand the behavior of the full system. Taking the singular limit of (1) yields the two-dimensional *fast subsystem* (*layer problem*)

2 $$ \textstyle\begin{cases} \dot{\mathbf{x}} = f(\mathbf{x},\mathbf{y},\lambda ), \\ \dot{\mathbf{y}} = 0, \end{cases} $$

where the slow variables **y** can be treated as bifurcation parameters. The flow of the fast subsystem is called the *fast flow*. In contrast, the *reduced system* (*slow subsystem*) is obtained by rescaling the time *t* of (1) to $\tau =\varepsilon t$ and then taking the singular limit. Hence, the reduced system is the two-dimensional system of differential algebraic equations

3 $$ \textstyle\begin{cases} 0 = f(\mathbf{x},\mathbf{y},\lambda ), \\ {y}^{\prime } = g(\mathbf{x},\mathbf{y},\lambda ), \end{cases} $$

where the prime denotes the derivative with respect to the slow time *τ*. The flow of the reduced system (3) is called the *slow flow*.

Equilibrium points of the fast subsystem (2) define a two-dimensional manifold called the *critical manifold*

4 $$ S:= \bigl\{ (\mathbf{x},\mathbf{y}) \in \mathbb{R}^{2} \times \mathbb{R}^{2} \mid f(\mathbf{x},\mathbf{y},\lambda )=0 \bigr\} , $$

which is also the phase space of the reduced system (3). A submanifold $S^{m} \subseteq S$ is *normally hyperbolic* if and only if all points on $S^{m}$ are hyperbolic equilibria with respect to the fast variables; that is, the eigenvalues of the Jacobian matrix $D_{\mathbf{x}}f(\mathbf{x},\mathbf{y},\lambda ,0)$ at these points have no zero real part. Depending on these eigenvalues, a corresponding submanifold $S^{m} \subseteq S$ can be attracting ($S^{a}$), repelling ($S^{r}$) or of saddle type ($S^{s}$). According to Fenichel theory, for *ε* sufficiently small, normally hyperbolic submanifolds of the critical manifold persist as locally invariant *slow manifolds* [11, 12]. A slow manifold $S^{m}_{\varepsilon }$ has the same smoothness and stability properties as the corresponding submanifold $S^{m} \subseteq S$. Moreover, the stable and unstable manifolds, $W^{s}(S^{m})$ and $W^{u}(S^{m})$, of a normally hyperbolic submanifold $S^{m}$ persist as locally invariant stable and unstable (fast) manifolds, $W^{s}(S^{m}_{\varepsilon })$ and $W^{u}(S^{m}_{\varepsilon })$, of the perturbed slow manifold $S^{m}_{\varepsilon }$, respectively. We also remark that the flow on the perturbed slow manifolds is diffeomorphic to the associated slow flow on the critical manifold for sufficiently small *ε*.

We are interested in the case where an attracting sheet $S^{a}$ meets a saddle sheet $S^{s}$ at a one-dimensional *fold curve* *F*. In this situation, the reduced system is singular (not normally hyperbolic) at *F*. Nevertheless, one attains a description of the dynamics near *F* by rescaling (3) to obtain the *desingularized reduced system*

5 $$ \textstyle\begin{cases} {\mathbf{x}}^{\prime } = \operatorname{adj}(D_{\mathbf{x}}f(\mathbf{x},\mathbf{y},\lambda )) \cdot D_{\mathbf{y}}f(\mathbf{x},\mathbf{y},\lambda ) \cdot g (\mathbf{x},\mathbf{y},\lambda ), \\ {\mathbf{y}}^{\prime } = -\operatorname{det}(D_{\mathbf{x}}f(\mathbf{x},\mathbf{y},\lambda )) \cdot g (\mathbf{x},\mathbf{y},\lambda ), \end{cases} $$

where $(\mathbf{x},\mathbf{y}) \in S$, and the prime now denotes the derivative with respect to $\tau _{1} = -\tau / \operatorname{det}(D_{\mathbf{x}}f(\mathbf{x},\mathbf{y},\lambda ))$. Here, $\operatorname{adj}(D_{\mathbf{x}}f(\mathbf{x},\mathbf{y},\lambda ))$ and $\operatorname{det}(D_{\mathbf{x}}f(\mathbf{x},\mathbf{y},\lambda ))$ are the adjoint and determinant of the Jacobian matrix $D_{\mathbf{x}}f(\mathbf{x},\mathbf{y},\lambda )$, respectively. Note that this rescaling causes a reversal of the flow on the saddle sheet $S^{s}$. Equilibrium points of the desingularized reduced system (5) that lie on fold curves are called *folded singularities*; they can be nodes, saddles or foci. In particular, the existence of a *folded node* on *F* allows for a whole sector of trajectories of (3) to pass from $S^{a}$ to $S^{s}$ in the singular limit $\varepsilon =0$. For *ε* sufficiently small, this sector gives rise to a finite number of *canard orbits*, i.e., trajectories that stay close to the attracting slow manifold $S^{a}_{\varepsilon }$ and saddle slow manifold $S^{s}_{\varepsilon }$ for $\mathcal{O}(1)$ time on the slow time scale [32–34].

## Computing Two-Dimensional Saddle Slow Manifolds

In this section, we introduce a general numerical framework for computing two-dimensional saddle slow manifolds and their stable and unstable manifolds in the context of systems with two fast and two slow variables. We also introduce a general method for computing and continuing canard orbits in this setting. We then illustrate our methods throughout with an extended version of the normal form of a folded node.

### Two-Point Boundary-Value Problem Setup for Four-Dimensional Slow–Fast Systems

To begin, we introduce a general setup for representing relevant parts of invariant manifolds in $\mathbb{R}^{4} $ as families of orbit segments by defining suitable 2PBVPs. Throughout the paper, we use this setup extensively to compute and continue invariant objects in systems of the form of (1). The first step for computing orbit segments as solutions of 2PBVPs is to rescale the vector field (1) as

6 $$ \dot{\mathbf{u}}=T H(\mathbf{u},\lambda ), $$

where $\mathbf{u} \in \mathbb{R}^{4} $ is the state, $\lambda \in \mathbb{R}^{p}$ is the parameter vector and $H: \mathbb{R}^{2} \times \mathbb{R}^{2} \times \mathbb{R}^{p} \times \mathbb{R} \mapsto \mathbb{R}^{4} $ is the right-hand side of (1). The parameter *T* is the total integration time of the computed orbit segment and it is treated as a free parameter; the overdot now represents the derivative with respect to the rescaled time $s=t/T$. Hence, the orbit segment $u(s)$ is always defined over the interval $s \in [0,1]$. We define codimension-*i* and codimension-*j* hypersurfaces, $\varXi _{0} $ and $\varXi _{1}$, where $i+j=4$ and then impose the boundary conditions

7 $$ \textstyle\begin{cases} \mathbf{u}(0) \in \varXi _{0}, \\ \mathbf{u}(1) \in \varXi _{1}. \end{cases} $$

Equation (6) with boundary conditions given by (7) yields a well-posed 2PBVP that defines a one-parameter solution family of orbit segments representing part of a two-dimensional manifold of interest. Furthermore, the solution family of this 2PBVP can be computed reliably by employing pseudo-arclength continuation in combination with a 2PBVP solver, such as collocation. A broader overview of this general 2PBVP setup can be found in [48].

Perhaps the simplest case of (7) is when $i=4$ and $j=0$, such that the corresponding 2PBVP effectively defines an initial value problem. For the case when $i=3$ and $j=1$, the 2PBVP yields orbit segments that start from a one-dimensional curve and end at a codimension-one submanifold. This case is commonly used to compute two-dimensional invariant manifolds as one-parameter families of orbit segments [48]. For systems of dimension higher than three, one-parameter families of orbit segments can also be computed by starting from a two-dimensional surface and ending at another two-dimensional surface. Implementation of the latter case, when $i=2$ and $j=2$, will be used to compute two-dimensional saddle slow manifolds.

2PBVP methods are very versatile and can be applied to compute and continue locally and globally invariant manifolds [31, 35, 45, 46, 53], global bifurcations [54–56], transient dynamics [21, 26, 57] and isochrons [58–61]. In this paper, we use various 2PBVP formulations to compute slow manifolds and their stable and unstable manifolds, as well as canard orbits; the respective families of solutions are found with the continuation software package AUTO [62].

### General Approach for Computing Two-Dimensional Slow Manifolds in $\mathbb{R}^{4}$

Computations of one-dimensional saddle slow manifolds and associated stable and unstable manifolds were performed with a collocation method and numerical integration [24], iterative methods [63] and two-point boundary-value problem (2PBVP) setup [21]. The flow on a one-dimensional saddle slow manifold is very simple because it has only one degree of freedom. In contrast, the flow on a two-dimensional saddle slow manifold may be quite intricate with subsets of trajectories that have different properties. We now present a general approach for computing suitable families of orbit segments to represent the relevant parts of two-dimensional attracting and saddle slow manifolds in $\mathbb{R}^{4}$. We consider a system of the form (1). We further assume that the critical manifold *S* of (1) has a two-dimensional attracting sheet $S^{a}$ and a two-dimensional saddle sheet $S^{s}$ that meet at a generic fold curve *F*. As was described in Sect. 3.1, we first rescale the vector field (1) to obtain (6), where the parameter *T* is the integration time associated with (1). We then implement a 2PBVP with the boundary conditions given in (7), where $\varXi _{0} $ and $\varXi _{1}$ are codimension-*i* and codimension-*j* hypersurfaces with $i+j=4$. The choice of $\varXi _{0} $ and $\varXi _{1}$ determines a one-parameter family of solutions that represent a given two-dimensional invariant manifold.

#### Computing Attracting and Repelling Slow Manifolds in $\mathbb{R}^{4}$

We compute the attracting slow manifold $S^{a}_{\varepsilon }$ in systems of the form (1) by using the boundary conditions

8 $$ \textstyle\begin{cases} u(0) \in L^{a}, \\ u(1) \in \varSigma ^{a} , \end{cases} $$

where $L^{a}$ is a one-dimensional curve on $S^{a}$ sufficiently away from *F*, and $\varSigma ^{a}$ is a codimension-one submanifold transverse to the flow. The computation of a repelling slow manifold can be performed in the same way by reversing time. This approach of computing attracting and repelling slow manifolds is a direct generalization to higher dimensions of the method used in [35, 38, 45, 48]; it can also be used for computing attracting and repelling slow manifolds in any system with two slow and an arbitrary number $n \geq 1$ of fast variables.

#### Computing Saddle Slow Manifolds in $\mathbb{R}^{4}$

Now we introduce a method for computing two-dimensional saddle slow manifolds in $\mathbb{R}^{4}$. This is a challenge because one needs to take into account the nature of the flow on the manifold. In addition, the existence of expanding and contracting fast normal directions means that a saddle slow manifold is not uniformly attracting in either forward or backward time. To deal with these issues, we define suitable 2PBVPs to compute different parts of a given saddle slow manifold. To set up the method, one needs some information about the behavior of the slow flow of the reduced system, as well as the stable and unstable eigendirections associated with the saddle sheet of the critical manifold in the singular limit.

We aim to represent the saddle slow manifold $S^{s}_{\varepsilon }$ in a system of the form (1) as a collection of one-parameter families of orbit segments that approach $S^{s}_{\varepsilon }$ along its stable manifold $W^{s}(S^{s}_{\varepsilon })$ and leave via its unstable manifold $W^{u}(S^{s}_{\varepsilon })$. This can be achieved by implementing suitable 2PBVPs. Specifically, we represent each part of $S^{s}_{\varepsilon }$ as a family of orbit segments satisfying the boundary conditions:

9 $$ \textstyle\begin{cases} u(0) \in \widetilde{\varSigma }_{0}, \\ u(1) \in \widetilde{\varSigma }_{1}. \end{cases} $$

Here, $\widetilde{\varSigma }_{0}$ and $\widetilde{\varSigma }_{1}$ are suitably chosen two-dimensional (codimension-two) sections. In our approach, each section $\widetilde{\varSigma }_{i}$ is defined as the intersection of two three-dimensional (codimension-one) submanifolds:

10 $$ \textstyle\begin{cases} \widetilde{\varSigma }_{0} := \widehat{\varSigma }_{0} \cap \varSigma _{0}, \\ \widetilde{\varSigma }_{1} := \widehat{\varSigma }_{1} \cap \varSigma _{1}. \end{cases} $$

Here, $\widehat{\varSigma }_{0}$ and $\widehat{\varSigma }_{1}$ are three-dimensional submanifolds that are chosen transverse to the stable manifold $W^{s}(S^{s}_{\varepsilon })$ and unstable manifold $W^{u}(S^{s}_{\varepsilon })$ of the saddle slow manifold $S^{s}_{\varepsilon }$, respectively; their definitions are informed by the (local) eigendirections of the equilibria of the fast subsystem that lie on $S^{s}$. In contrast, the three-dimensional submanifolds $\varSigma _{0}$ and $\varSigma _{1}$ are constraints that are tailored to the desired submanifold of $S^{s}_{\varepsilon }$ to be computed. In this way, we can represent a suitable submanifold of $S^{s}_{\varepsilon }$ as a one-parameter family of orbit segments that enter along $W^{s}(S^{s}_{\varepsilon })$, and then stay extremely close to $S^{s}_{\varepsilon }$ before exiting along $W^{u}(S^{s}_{\varepsilon })$.

### Extended Normal Form of a Folded Node

Our approach of computing two-dimensional saddle slow manifolds is general, but requires some knowledge of the slow flow on the critical manifold. To illustrate it, we now introduce a concrete example of a four-dimensional vector field to provide a case study. The normal form of a folded node is a system with one fast and two slow variables [34, 47]. Here, we introduce and consider the extended vector field given by

11 $$ \textstyle\begin{cases} \dot{x} = \varepsilon (\frac{1}{2} \mu y - (\mu +1) z ), \\ \dot{y} = \varepsilon , \\ \dot{z} = x+ z^{2}, \\ \dot{w} = z-w, \end{cases} $$

which includes a second fast variable *w* representing a stable fast direction. Since *w* does not play a role in the dynamics of the other three variables, the dynamics of *x*, *y* and *z* of the original model, including the slow flow on the critical manifold, remains unchanged. Hence, this system is a good test-case model for computing two-dimensional saddle slow manifolds in $\mathbb{R}^{4}$. The critical manifold of (11) is given by the two-dimensional parabolic surface

12 $$ S:= \bigl\{ (x,y,z,w) \mid x=- z^{2}, \mbox{ and } z=w \bigr\} , $$

which has an attracting sheet $S^{a}$ and a saddle sheet $S^{s}$ that meet at a nondegenerate fold curve *F*. More precisely, *S* is composed of

13 $$ \textstyle\begin{cases} S^{a} := \{ (x,y,z,w) \in S \mid z< 0 \} , \\ F := \{ (x,y,z,w) \in S \mid z=0 \} , \\ S^{s} := \{ (x,y,z,w) \in S \mid z>0 \} . \end{cases} $$

The reduced system of (11) is given by

14 $$ \textstyle\begin{cases} x^{\prime } = \frac{1}{2} \mu y - (\mu +1) z, \\ y^{\prime } = 1, \\ 0 = x+ z^{2}, \\ 0 = z-w. \end{cases} $$

It defines the slow flow, where the prime denotes the derivative with respect to the slow time scale $\tau = \varepsilon t$. The slow flow on the saddle sheet $S^{s}$ is the image of the slow flow on $S^{a}$ under the symmetry

15 $$ (x, y, z, w) \mapsto (x,-y,-z, -w). $$

The origin is a folded singularity, which is an equilibrium of the desingularized reduced system on *F* but not of the full system. Its eigenvalues as an equilibrium of the corresponding desingularized reduced system are −*μ* and −1; thus, the folded singularity is a node for $\mu >0$. In the calculations that follow, we first consider $\mu =9.2$.

**Fig. 1:**
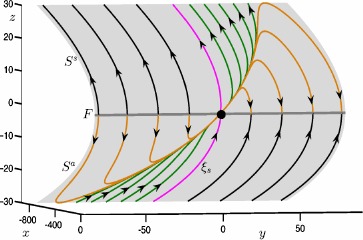
Critical manifold of (11) with the phase portrait of the reduced system (14) with $\mu =9.2$, projected onto the ($x,y,z$)-space. The lower sheet $S^{a}$ of the critical manifold (gray) is attracting and the upper sheet $S^{s}$ is of saddle type. The fold curve *F* (gray line) separates the two sheets. The black dot at the origin is the folded node, and the magenta curve $\xi _{s}$ is the strong singular canard

Figure 1 illustrates the slow flow in the neighborhood of the folded node (black dot) on the critical manifold (gray surface), which is projected onto the ($x,y,z$)-space. The fold curve *F* (gray line) separates the attracting sheet $S^{a}$ and the saddle sheet $S^{s}$ of the critical manifold. The magenta trajectory is the strong singular canard $\xi _{s}$, which divides $S^{a}$ into a funnel region and a jump region; see [34] for details. There are three types of trajectories on $S^{a}$ and $S^{s}$ that behave differently. Orange and green trajectories on $S^{a}$, which lie in the funnel region between $\xi _{s}$ and *F*, move to $S^{s}$ through the folded node in finite time. The black trajectories on $S^{a}$ move towards *F* and then jump in the fast direction. The black and green trajectories on $S^{s}$ move away from *F* all the way to infinity. On the other hand, orange trajectories on $S^{s}$ leave from the vicinity of the folded node and make a return to the fold curve *F*, where they jump in the fast direction.

### Slow Manifolds of the Extended Normal Form

Here, we illustrate our approach of computing attracting and saddle slow manifolds with system (11). Since the flow on the attracting slow manifold $S^{a}_{\varepsilon }$ and the saddle slow manifold $S^{s}_{\varepsilon }$, for *ε* small enough, is diffeomorphic to that on $S^{a}$ and $S^{s}$, respectively, we use the nature of the slow flow on the respective parts of the critical manifold shown in Fig. 1 as a guide for selecting families of orbit segments that represent the slow manifolds. With this selection, we compute the slow manifolds with the general approach introduced in Sect. 3.2.

#### Computing the Saddle Slow Manifold of the Normal Form

We represent the saddle slow manifold $S^{s}_{\varepsilon }$ of (11) as families of orbit segments that satisfy the boundary conditions given by (9) and (10). The three-dimensional submanifolds $\widehat{\varSigma }_{0}$ and $\widehat{\varSigma }_{1}$ of (10) need to be transverse to the stable manifold $W^{s}(S^{s}_{\varepsilon })$ and unstable manifold $W^{u} (S^{s}_{\varepsilon })$ of the saddle slow manifold $S^{s}_{\varepsilon }$, respectively. The extended normal form has the special property that each equilibrium of the fast subsystem on $S^{s}$ has the *w*-direction as its stable eigendirection, whereas the unstable eigendirection is defined by $(x,z)=(2z+1,1)$. This property implies that, away from *F*, trajectories on $W^{s}(S^{s}_{\varepsilon })$ always enter $S^{s}_{\varepsilon }$ via the *w*-nullcline, and trajectories on $W^{u}(S^{s}_{\varepsilon })$ always move away from $S^{s}_{\varepsilon }$ via the *z*-nullcline. Hence, we choose

16 $$ \textstyle\begin{cases} \widehat{\varSigma }_{0} := \{ (x,y,z,w) \mid \dot{w} =0 \} = \{ (x,y,z,w) \mid w=z \} , \\ \widehat{\varSigma }_{1} := \{ (x,y,z,w) \mid \dot{z} =0 \} = \{ (x,y,z,w) \mid x=-z^{2} \} , \end{cases} $$

which guarantees that $\widehat{\varSigma }_{0}$ and $\widehat{\varSigma }_{1}$ are transverse to $W^{s}(S^{s}_{\varepsilon })$ and $W^{u}(S^{s}_{\varepsilon })$, respectively. We use these choices for $\widehat{\varSigma }_{0}$ and $\widehat{\varSigma }_{1}$ for all computed submanifolds of $S^{s}_{\varepsilon }$. On the other hand, sections $\varSigma _{0}$ and $\varSigma _{1}$ determine the constraints that specify the computed submanifolds of $S^{s}_{\varepsilon }$. They are different for each of the three families of orbit segments shown in Fig. 1. More specifically, the submanifold of $S^{s}_{\varepsilon }$ corresponding to the black orbit segments in Fig. 1 is specified by

17 $$ \textstyle\begin{cases} \varSigma _{0} := \{ (x,y,z,w) \mid z=0.1 \} , \\ \varSigma _{1} := \{ (x,y,z,w) \mid z=30 \} , \end{cases} $$

so that we capture the start of these orbit segments near the fold curve *F* and follow them up to $z=30$. The submanifold of $S^{s}_{\varepsilon }$ corresponding to the green orbit segments is specified by

18 $$ \textstyle\begin{cases} \varSigma _{0} := \{ (x,y,z,w) \mid y=0 \} , \\ \varSigma _{1} := \{ (x,y,z,w) \mid z=30 \} , \end{cases} $$

because these orbit segments all start at $y=0$ near the folded node. Finally, the submanifold of $S^{s}_{\varepsilon }$ corresponding to the orange orbit segments is specified by

19 $$ \textstyle\begin{cases} \varSigma _{0} := \{ (x,y,z,w) \mid y=0 \} , \\ \varSigma _{1} := \{ (x,y,z,w) \mid z=0.1 \} , \end{cases} $$

because these orbits segments end close to *F*. The 2PBVPs of the three submanifolds that constitute the computed saddle slow manifold are defined by Eq. (6) with boundary conditions (9), (10) as specified by (16), and one of (17), (18) or (19).

**Fig. 2:**
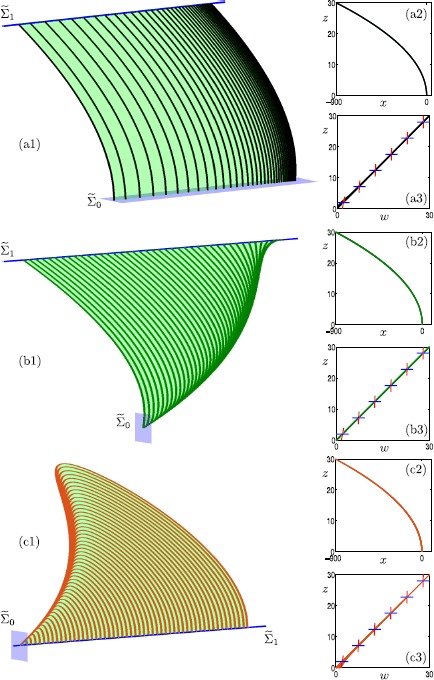
Three computed submanifolds of $S^{s}_{\varepsilon }$ of system (11) with $\mu =9.2$ and $\varepsilon =0.01$. The left column illustrates the three submanifolds, projected onto the ($x,y,z$)-space, together with the corresponding boundary conditions $\widetilde{\varSigma }_{0}$ and $\widetilde{\varSigma }_{1}$ (light- and dark-blue sections). The right column shows the three submanifolds, projected onto the ($x,z$)-plane and the ($w,z$)-plane, where we also include stable (blue) and unstable (red) eigenvector directions at a selection of points on $S^{s}$

Figure 2 shows the three computed submanifolds of the saddle slow manifold $S^{s}_{\varepsilon }$ for $\varepsilon =0.01$. They correspond to the three different families of trajectories on $S^{s}$ shown in Fig. 1. The left column shows each submanifold, projected onto the ($x,y,z$)-space. The sections $\widetilde{\varSigma }_{0}$ (light blue) and $\widetilde{\varSigma }_{1}$ (dark blue) used for the boundary conditions are also shown. The right column of Fig. 2 shows the same submanifolds of $S^{s}_{\varepsilon }$, projected onto the ($x,z$)- and ($w,z$)-planes. In these projections, the saddle branch $S^{s}$ of the critical manifold correspond to $\{ (x,z) \mid x=-z^{2} \text{ and } z>0 \} $ and $\{ (w,z) \mid z=w \mbox{ and } z>0 \} $, respectively. In both projections, it is notable that all computed orbit segments of $S^{s}_{\varepsilon }$ stay extremely close to $S^{s}$; this is an indication that they give a good approximation of the saddle slow manifold. In the ($w,z$)-plane, the short blue (red) segments are the directions of the stable (unstable) eigenvectors associated with the equilibrium points of $S^{s}$ at the singular limit $\varepsilon =0$.

Figure 2(a) shows the computed orbit segments (black) of the first family of $S^{s}_{\varepsilon }$. The green surface, which is a part of $S^{s}_{\varepsilon }$, has been rendered from these black orbit segments. Indeed, the black family of orbit segments are topologically as the black trajectories on $S^{s}$ shown in Fig. 1. In Fig. 2(a1), the sections $\widetilde{\varSigma }_{0}$ (light-blue plane) and $\widetilde{\varSigma }_{1}$ (dark-blue line) correspond to the boundary conditions that define the computed black orbit segments. The section $\widetilde{\varSigma }_{1}$ is actually two dimensional but it appears as a one-dimensional line in projection onto the ($x,y,z$)-space because the corresponding equation does not depend on *w*. The boundary conditions for the shown submanifold of $S^{s}_{\varepsilon }$ are defined by (9), (10), (16), and (17). As a result, the computed family of orbit segments start from the vicinity of the fold curve *F* and end far away from *F*. Panels (a2) and (a3) of Fig. 2 show that this family of $S^{s}_{\varepsilon }$ is very close to $S^{s}$.

Observe from Fig. 2(a3) that the stable eigenvectors are aligned with the *w*-axis. Also note that, sufficiently far away from *F*, the unstable eigenvectors are (almost) aligned with the *z*-axis. These eigendirections illustrate why we force the computed orbit segments representing this part of $S^{s}_{\varepsilon }$ to start from the *w*-nullcline and end at the *z*-nullcline; see (16). In this way, we compute orbit segments of $S^{s}_{\varepsilon }$ without including the fast segments of the associated stable and unstable manifolds. However, as we will show in Sect. 3.5, we are able to change the boundary conditions and extend the orbit segments, in backward and forward time, to include fast segments of $W^{s}(S^{s}_{\varepsilon })$ and $W^{u}(S^{s}_{\varepsilon })$, respectively.

Figure 2(b) shows the computed orbit segments (green) of the second family of $S^{s}_{\varepsilon }$. This family is topologically as the green family of trajectories on $S^{s}$ shown in Fig. 1. The green surface, which is a part of $S^{s}_{\varepsilon }$, has been rendered from the shown green orbit segments. Sections $\widetilde{\varSigma }_{0}$ (light-blue plane) and $\widetilde{\varSigma }_{1}$ (dark-blue line) illustrate the respective boundary conditions. Here, the section $\widetilde{\varSigma }_{1}$ is also two dimensional but it again appears as a one-dimensional curve in projection onto the ($x,y,z$)-space. The 2PBVP setup for this submanifold of $S^{s}_{\varepsilon }$ is defined by (9), (10), (16), and (18). The green orbit segments start from the neighborhood of the folded node and terminate sufficiently far away from the fold curve. In both projections shown in panels (b2) and (b3), the computed orbit segments lie extremely close to $S^{s}$, which indicates that the computed orbit segments again provide a good approximation of the saddle slow manifold.

Finally, Fig. 2(c) shows the computed orbit segments (orange) of the third family of $S^{s}_{\varepsilon }$. The boundary conditions for this submanifold are (9), (10), (16) and (19). The corresponding orbits segments start from the neighborhood of the folded node and make a return to the fold curve *F*. Panel (c3) shows that the orbit segments divert slightly from the critical manifold *S* as they return to the vicinity of *F*. This is because the linearization of the unstable manifold $W^{u}(S^{s})$, which is given by $(z,w)=(1+2z,1)$, becomes more aligned with the slow flow on the critical manifold as *F* is approached. In other words, the normal hyperbolicity of the saddle slow manifold is lost near *F*. Nevertheless, the projections in panels (c2) and (c3) indicate that the computed orbit segments still provide a good approximation of the saddle slow manifold.

#### The Overall Geometry of Slow Manifolds

As was described in Sect. 3.2, we compute the attracting slow manifold $S^{a}_{\varepsilon }$ by starting from a line away from the fold curve *F* and ending at a three-dimensional submanifold. More specifically, we represent $S^{a}_{\varepsilon }$ as families of orbit segments that are subject to the boundary condition given by (8), where the precise loci of $L^{a}$ and $\varSigma ^{a}$ depend on the desired submanifold of $S^{a}_{\varepsilon }$ to be computed. To represent the black, green and orange trajectories of $S^{a}$ in Fig. 1 as orbit segments of $S^{a}_{\varepsilon }$, we choose

20 $$\begin{gathered} \textstyle\begin{cases} u(0) \in L^{a}_{1} := \{ (x,y,z,w) \mid z=-30 \} \cap S^{a}, \\ u(1) \in \varSigma ^{a}_{1} := \{ (x,y,z,w) \mid z=-0.1 \} , \end{cases}\displaystyle \end{gathered}$$

21 $$\begin{gathered} \textstyle\begin{cases} u(0) \in L^{a}_{1} := \{ (x,y,z,w) \mid z=-30 \} \cap S^{a}, \\ u(1) \in \varSigma ^{a}_{2} := \{ (x,y,z,w) \mid y=0 \} , \end{cases}\displaystyle \end{gathered}$$

and

22 $$ \textstyle\begin{cases} u(0) \in L^{a}_{2} := \{ (x,y,z,w) \mid z=-0.1 \} \cap S^{a}, \\ u(1) \in \varSigma ^{a}_{2} := \{ (x,y,z,w) \mid y=0 \} , \end{cases} $$

respectively. These boundary conditions result in three families of orbit segments that correspond to three submanifolds constituting the attracting slow manifold $S^{a}_{\varepsilon }$.

**Fig. 3:**
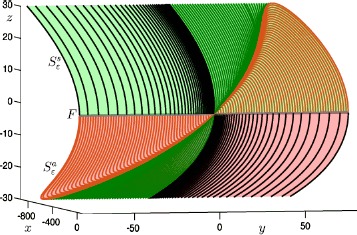
Attracting slow manifold $S^{a}_{\varepsilon }$ (red surface) and saddle slow manifold $S^{s}_{\varepsilon }$ (green surface), projected onto $(x,y,z)$-space, for system (11) with $\mu =9.2$ and $\varepsilon =0.01$. The gray line is the fold curve *F* of the critical manifold. These surfaces were rendered from the orbit segments shown; compare with Fig. 2

Figure 3 shows the resulting approximations of the attracting slow manifold $S^{a}_{\varepsilon }$ (red surface) and the saddle slow manifold $S^{s}_{\varepsilon }$ (green surface) for $\varepsilon =0.01$; also shown are the computed orbit segments used to render the surfaces. Note that the flow on the slow manifolds is qualitatively the same as the slow flow of the reduced system ($\varepsilon =0$) shown in Fig. 1, as one may expect for such a small value of $\varepsilon =0.01$. The saddle slow manifold $S^{s}_{\varepsilon }$ is rendered as a concatenation of the three families of orbit segments as shown in Fig. 2. Likewise, the attracting slow manifold $S^{a}_{\varepsilon }$ is rendered as a concatenation of the three computed families of orbit segments based on the boundary conditions (20), (21) and (22), respectively. The flow on $S^{a}_{\varepsilon }$ is topologically equivalent to the slow flow on $S^{a}$ in Fig. 1. Black trajectories of $S^{a}_{\varepsilon }$ approach the fold curve *F*, near which they are expected to make a jump away from the critical manifold. In contrast, green and orange trajectories of $S^{a}_{\varepsilon }$ terminate near the folded node where they interact with the saddle slow manifold. The interaction between $S^{a}_{\varepsilon }$ and $S^{s}_{\varepsilon }$ will be discussed in Sect. 4.

### Computing Stable and Unstable Manifolds of Saddle Slow Manifolds

Two-dimensional saddle slow manifolds in $\mathbb{R}^{4}$ are associated with three-dimensional stable and unstable (fast) manifolds. It is very challenging to compute and visualize such three-dimensional geometric objects. Nevertheless, we now present a general approach for computing two-dimensional submanifolds of the three-dimensional stable manifold $W^{s}(S^{s}_{\varepsilon })$ and the three-dimensional unstable manifold $W^{u}(S^{s}_{\varepsilon })$ of the saddle slow manifold $S^{s}_{\varepsilon }$. This can be achieved by implementing suitable 2PBVPs to compute orbit segments that start transversally to $W^{s}(S^{s}_{\varepsilon })$ and end transversally to $W^{u}(S^{s}_{\varepsilon })$. The basic idea of our approach is inspired by the computation of two-dimensional (un)stable manifolds of a one-dimensional saddle slow manifold, which is presented in [21].

Consider system (1) and assume that it features a two-dimensional saddle slow manifold $S^{s}_{\varepsilon }$ with associated stable manifold $W^{s}(S^{s}_{\varepsilon })$ and unstable manifold $W^{u}(S^{s}_{\varepsilon })$. Each normally hyperbolic trajectory $c^{s}_{\varepsilon } \subset S^{s}_{\varepsilon }$ on the saddle slow manifold is associated with two-dimensional stable and unstable manifolds, $W^{s}(c^{s}_{\varepsilon })$ and $W^{u}(c^{s}_{\varepsilon })$, that approach the (slow) trajectory $c^{s}_{\varepsilon }$ at an exponential rate in forward and backward time, respectively. To compute the stable manifold $W^{s}(c^{s}_{\varepsilon })$ of a trajectory $c^{s}_{\varepsilon }$, we first extend $c^{s}_{\varepsilon }$ by the flow in backward time to include a fast stable segment. This can be done for each of the two sides of the corresponding stable eigenvectors. We then continue the resulting extended orbit segment as a one-parameter family of orbit segments subject to the boundary conditions

23 $$ \textstyle\begin{cases} u(0) \in \varSigma ^{s}, \\ u(1) \in L^{s} , \end{cases} $$

where $\varSigma ^{s}$ is a codimension-one submanifold far away from *S* and transverse to the stable manifold of the trajectory $c^{s}_{\varepsilon }$. On the other hand, $L^{s}$ is a line segment close to the saddle sheet $S^{s}$ and transverse to the unstable manifold of the trajectory $c^{s}_{\varepsilon }$.

We can also compute two-dimensional submanifolds of the stable manifold $W^{s}(S^{s}_{\varepsilon })$ associated with a one-dimensional curve on $S^{s}_{\varepsilon }$. This can be achieved by imposing the boundary conditions

24 $$ \textstyle\begin{cases} u(0) \in \varSigma ^{s}_{0}, \\ u(1) \in \varSigma ^{s}_{1}, \end{cases} $$

where $\varSigma ^{s}_{1}$ and $\varSigma ^{s}_{0}$ are codimension-two submanifolds that are suitably chosen to be transverse to $W^{s}(S^{s}_{\varepsilon })$ and $W^{u}(S^{s}_{\varepsilon })$, respectively.

Submanifolds of the unstable manifold $W^{u}(S^{s}_{\varepsilon })$ can be computed with the same setup after reversing time. The precise definition of the 2PBVPs given by (23) and (24) depends on the desired computed submanifold, as will be described for our specific example in the next section.

#### Stable and Unstable Manifolds of a Trajectory on $S^{s}_{\varepsilon }$

**Fig. 4:**
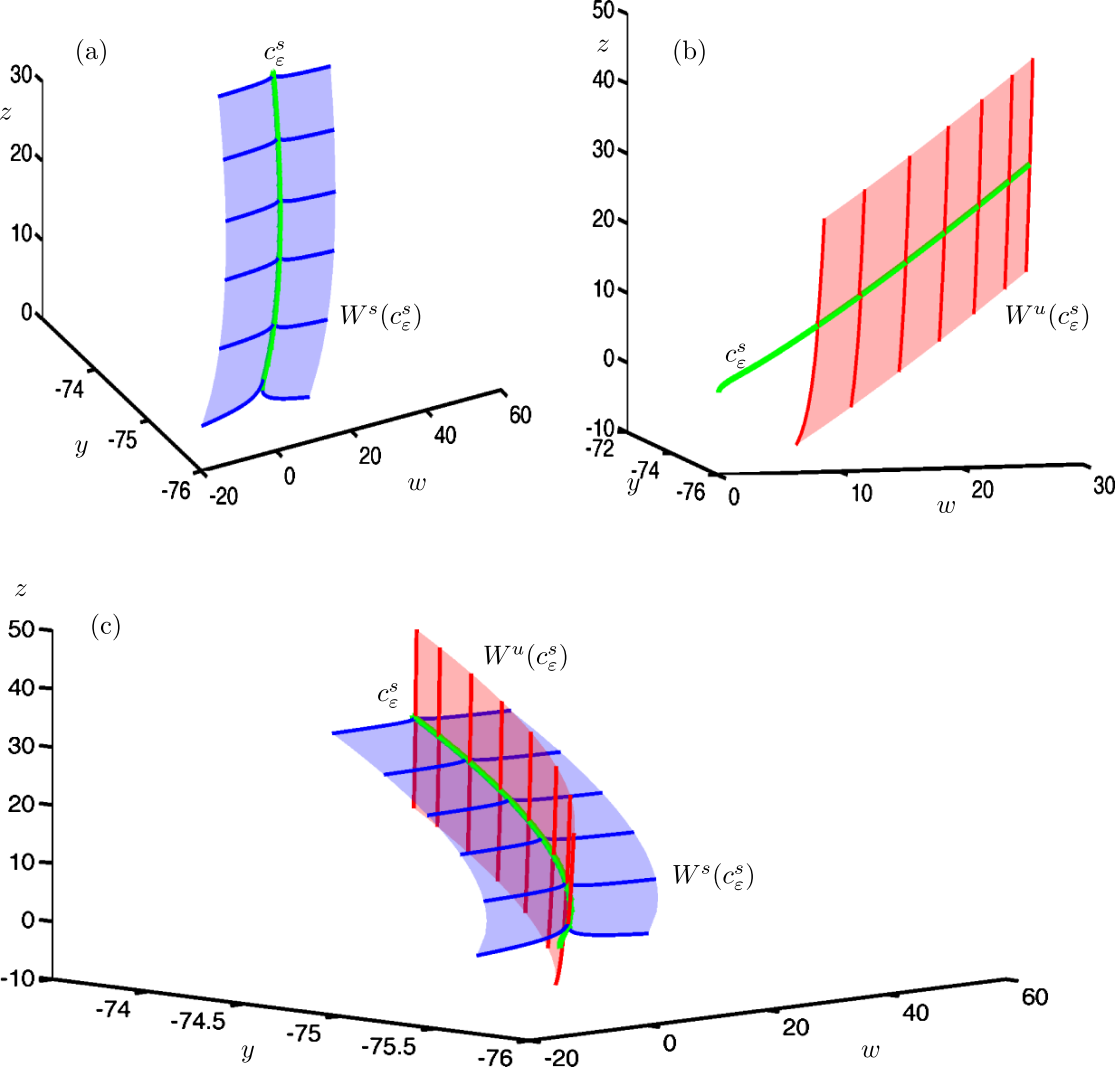
A trajectory $c^{s}_{\varepsilon }$ (green) on the saddle slow manifold $S^{s}_{\varepsilon }$ and its stable manifold $W^{s}(c^{s}_{\varepsilon })$ (blue) and unstable manifold $W^{s}(c^{s}_{\varepsilon })$ (red) in system (11) with $\mu =9.2$ and $\varepsilon =0.01$. A selection of orbit segments of $W^{s}(c^{s}_{\varepsilon })$ and $W^{s}(c^{s}_{\varepsilon })$ are shown as blue and red thick curves, respectively. Panel **(a)** shows the stable manifold $W^{s}(c^{s}_{\varepsilon })$, panel **(b)** shows the unstable manifold $W^{u}(c^{s}_{\varepsilon })$ and panel **(c)** shows both

Figure 4 shows the stable manifold $W^{s}(c^{s}_{\varepsilon })$ (blue surfaces) and unstable manifold $W^{u}(c^{s}_{\varepsilon })$ (red surfaces) of a selected trajectory $c^{s}_{\varepsilon }$ (green curve) on the saddle slow manifold $S^{s}_{\varepsilon }$, projected onto the $(w,y,z)$-space. Figure 4(a) shows an approximation of the two-dimensional local manifold $W^{s}(c^{s}_{\varepsilon })$ (blue surface); also shown is a selection of orbit segments on $W^{s}(c^{s}_{\varepsilon })$ (blue curves). Each side of the local manifold is computed by starting from a chosen orbit segment, which is then continued as a one-parameter family that satisfies the boundary conditions

25 $$ \textstyle\begin{cases} u(0) \in \varSigma ^{s} := \{ (x,y,z,w) \mid z=w \} , \\ u(1) \in L^{s} := \{ (x,y,z,w) \mid x=-z^{2}; z=30; y=-73.8301 \} . \end{cases} $$

Here, $\varSigma ^{s}$ is a codimension-one submanifold and $L^{s}$ is a one-dimensional curve. The submanifold $\varSigma ^{s}$ is chosen to be transverse to $W^{s}(c^{s}_{\varepsilon })$ and sufficiently far away from *S*. The curve $L^{s}$ is chosen to be transverse to $W^{u}(c^{s}_{\varepsilon })$. This computational setup forces the solution segments to move along $W^{s}(c^{s}_{\varepsilon })$ before converging to the slow trajectory $c^{s}_{\varepsilon }$ (in the direction of increasing *z*).

Figure 4(b) shows the local manifold $W^{u}(S^{s}_{\varepsilon })$ (red surfaces) of the same trajectory $c^{s}_{\varepsilon }$ (green curve), together with a selection of orbit segments on $W^{u}(c^{s}_{\varepsilon })$ (red curves). Again, we compute each side of the local manifold by starting from a chosen orbit segment, which is then continued as a one-parameter family that satisfies the boundary conditions

26 $$ \textstyle\begin{cases} u(0) \in L^{u} := \{ (x,y,z,w) \mid z=w;z=1; y=-75.5057 \} , \\ u(1) \in \varSigma ^{u} := \{ (x,y,z,w) \mid x=-z^{2} \} , \end{cases} $$

where $L^{u}$ and $\varSigma ^{u}$ are chosen to be transverse to $W^{s}(c^{s}_{\varepsilon })$ and $W^{u}(c^{s}_{\varepsilon })$, respectively. The computed orbit segments move along the slow trajectory $c^{s}_{\varepsilon }$ (again in the direction of increasing *z*), before exiting via its two-dimensional unstable manifold $W^{u}(c^{s}_{\varepsilon })$.

Figure 4(c) shows both $W^{s}(c^{s}_{\varepsilon })$ (blue) and $W^{u}(c^{s}_{\varepsilon })$ (red) of the same trajectory $c^{s}_{\varepsilon }$ (green). Note that they are indeed aligned with the *w*- and *z*-axes, respectively; this is consistent with the directions of the associated eigenvectors of the saddle sheet $S^{s}$, as expected for small $\varepsilon =0.01$; see Fig. 2.

#### Slices of Stable and Unstable Manifolds of $S^{s}_{\varepsilon }$

**Fig. 5:**
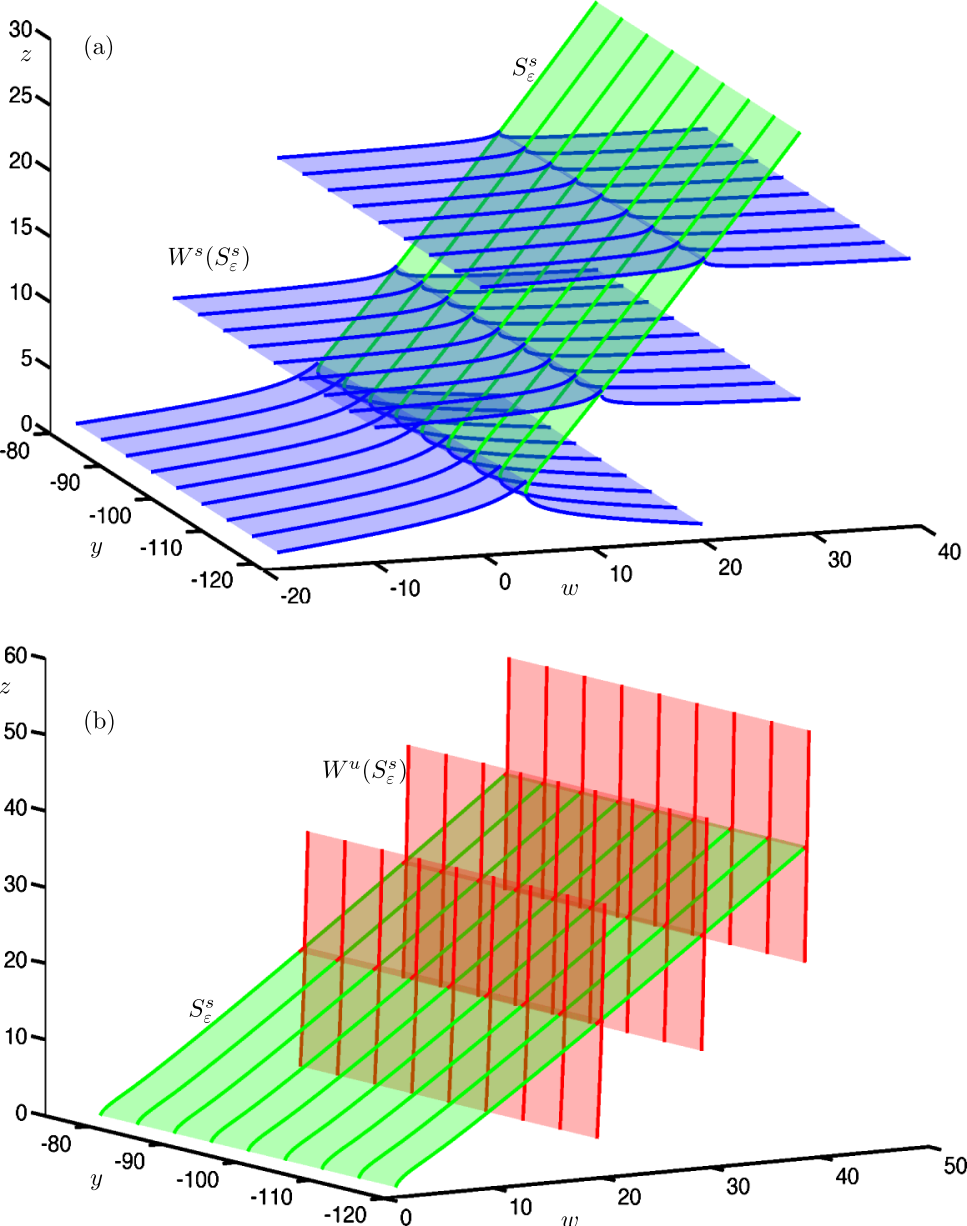
A selection of two-dimensional submanifolds of the three-dimensional manifolds $W^{s}(S^{s}_{\varepsilon })$ and $W^{u}(S^{s}_{\varepsilon })$ of system (11) with $\mu =9.2$ and $\varepsilon =~0.01$. Panel **(a)** shows a piece of $S^{s}_{\varepsilon }$ (green surface) and associated two-dimensional submanifolds of $W^{s}(S^{s}_{\varepsilon })$ (blue surface). Panel **(b)** shows a piece of $S^{s}_{\varepsilon }$ (green surface) and associated two-dimensional submanifolds of $W^{u}(S^{s}_{\varepsilon })$ (red surface). All surfaces are rendered from the respective thick orbit segments

To compute different parts of the three-dimensional manifolds $W^{s}(S^{s}_{\varepsilon })$ and $W^{u}(S^{s}_{\varepsilon })$, we implement variations of suitable 2PBVPs. Figure 5 shows a two-dimensional piece of $S^{s}_{\varepsilon }$ (green surface), together with six two-dimensional submanifolds of the associated three-dimensional stable manifold $W^{s}(S^{s}_{\varepsilon })$ (blue surfaces) and unstable manifold $W^{u}(S^{s}_{\varepsilon })$ (red surfaces) of system (11). All surfaces are rendered from the respective computed orbit segments (thick curves).

Figure 5(a) shows six two-dimensional submanifolds of $W^{s}(S^{s}_{\varepsilon })$, each formed by a collection of particular orbit segments on $W^{s}(c^{s}_{\varepsilon })$ for a family of slow trajectories $c^{s}_{\varepsilon }$ on $S^{s}_{\varepsilon }$. More precisely, each submanifold corresponds to a one-parameter family of orbit segments that satisfy the boundary conditions

27 $$ \textstyle\begin{cases} u(0) \in \varSigma ^{s}_{0}:= \{ (x,y,z,w) \mid \dot{w} =0 \} \cap \{ (x,y,z,w) \mid w=w_{s} \} , \\ u(1) \in \varSigma ^{s}_{1}:= \{ (x,y,z,w) \mid \dot{z} =0 \} \cap \{ (x,y,z,w) \mid z=30 \} , \end{cases} $$

where the sections $\varSigma ^{s}_{0}$ and $\varSigma ^{s}_{1}$ are two-dimensional constraints that are chosen to be transverse to $W^{s}(S^{s}_{\varepsilon })$ and $W^{u}(S^{s}_{\varepsilon })$, respectively, and $w_{s} $ is a suitably chosen constant that identifies a particular selection of orbit segments on $W^{s}(S^{s}_{\varepsilon })$. The six selected submanifolds of $W^{s}(S^{s}_{\varepsilon })$ are defined by $w_{s} =-19, -10,0,20,30$, and 40.

Similarily, Fig. 5(b) shows six two-dimensional submanifolds of the three-dimensional unstable manifold $W^{u}(S^{s}_{\varepsilon })$. Each submanifold corresponds to a one-parameter family of orbit segments that satisfy the boundary conditions

28 $$ \textstyle\begin{cases} u(0) \in \varSigma ^{u}_{0}:= \{ (x,y,z,w)\mid \dot{w} =0 \} \cap \{ (x,y,z,w)\mid w=1 \} , \\ u(1) \in \varSigma ^{u}_{1}:= \{ (x,y,z,w)\mid \dot{z} =0 \} \cap \{ (x,y,z,w)\mid z=z_{u} \} , \end{cases} $$

where $\varSigma ^{u}_{0}$ and $\varSigma ^{u}_{1}$ are transverse to $W^{s}(S^{s}_{\varepsilon })$ and $W^{u}(S^{s}_{\varepsilon })$, respectively. The six shown submanifolds of $W^{s}(S^{s}_{\varepsilon })$ are selected by setting $z_{u} =5,15,25,35.7, 45.5$, and 55.4, which again specifies the different two-dimensional submanifolds of $W^{u}(S^{s}_{\varepsilon })$ as particular selections of orbit segments on $W^{u}(S^{s}_{\varepsilon })$ for a family of trajectories $c^{s}_{\varepsilon }$ on $S^{s}_{\varepsilon }$.

## Interaction Between Attracting and Saddle Slow Manifolds and Canard Orbits in $\mathbb{R}^{4}$

Slow manifolds can be extended by the flow up to a transverse section near a folded node where they interact with each other. In systems of ordinary differential equations with one fast and two slow variables, extended attracting and repelling slow manifolds may intersect transversally in isolated canard orbits. In such systems, canard orbits can be detected by placing a cross-section transverse to both manifolds near the folded node and determining their intersections; see also [35, 45, 48, 64]. In systems with two fast and two slow variables, attracting and saddle slow manifolds spiral around each other in forward and backward time, respectively, in the vicinity of the folded node. However, their possible intersections do not occur in a structurally stable manner in $\mathbb{R}^{4}$. We now investigate the interaction between two-dimensional attracting and saddle slow manifolds of the four-dimensional system (11). In the computations that follow, we set $\mu =100.1$ in order to obtain more interesting dynamics near the folded node, that is, more canard orbits near $\varepsilon =0$.

**Fig. 6:**
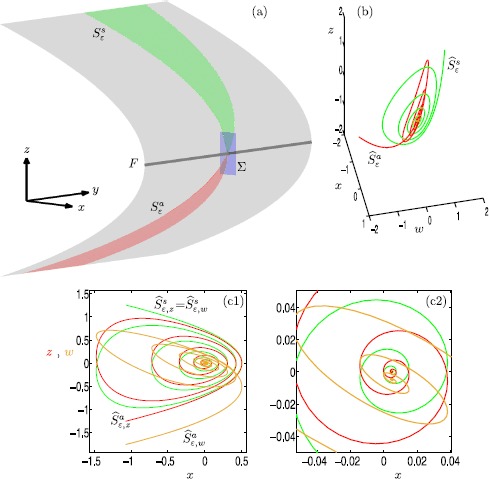
Interaction between $S^{a}_{\varepsilon }$ and $S^{s}_{\varepsilon }$ in system (11) with $\mu =100.1$ and $\varepsilon =0.01$. Panel **(a)** shows $S^{a}_{\varepsilon }$ (red) and $S^{s}_{\varepsilon }$ (green) projected onto the $(x,y,z)$-space, and computed up to the three-dimensional section *Σ* (blue); the gray surface is the critical manifold *S* and the gray line is the fold curve *F*. Panel **(b)** shows the intersection curves $\widehat{S}^{a}_{\varepsilon } = S^{a}_{\varepsilon } \cup \varSigma $ (red) and $\widehat{S}^{s}_{\varepsilon } = S^{s}_{\varepsilon } \cup \varSigma $ (green) in the ($x,w,z$)-space of *Σ*. Panel **(c1)** shows the curves $\widehat{S}^{a}_{\varepsilon ,z}$ (red) and $\widehat{S}^{a}_{\varepsilon ,w}$ (orange) as projections of $\widehat{S}^{a}_{\varepsilon }$ onto the $(x,z)$- and $(x,w)$-planes, respectively, together with the intersection curve $\widehat{S}^{s}_{\varepsilon }$ (green), which is the same in both projections. Panel **(c2)** is an enlargement of panel **(c1)**

Figure 6 illustrates how the attracting slow manifold $S^{a}_{\varepsilon }$ and saddle slow manifold $S^{s}_{\varepsilon }$ interact with each other near the folded node (the origin) in system (11) with $\mu =100.1$ and $\varepsilon =0.01$. Panel (a) shows a submanifold of $S^{a}_{\varepsilon }$ (red surface) and a submanifold of $S^{s}_{\varepsilon }$ (green surface), computed up to the three-dimensional section $\varSigma := \{ y=0 \} $ (blue plane), which contains the folded node at the origin; the figure shows a projection onto the ($x,y,z$)-space. Each surface intersects *Σ* in a one-dimensional curve. Panel (b) shows the intersection curves $\widehat{S}^{a}_{\varepsilon } = S^{a}_{\varepsilon } \cup \varSigma $ (red) and $\widehat{S}^{s}_{\varepsilon } = S^{s}_{\varepsilon } \cup \varSigma $ (green) in the ($x,w,z$)-space representing *Σ*. The two curves spiral around each other but do not actually intersect. Panel (c1) illustrates the same two curves in simultaneous projections onto the ($x,z$)- and ($x,w$)-planes; and panel (c2) is an enlargement. The curves $\widehat{S}^{a}_{\varepsilon ,z}$ (red) and $\widehat{S}^{a}_{\varepsilon ,w}$ (orange) are the projections of $\widehat{S}^{a}_{\varepsilon }$ onto the ($x,z$)- and ($x,w$)-planes, respectively. The curve $\widehat{S}^{s}_{\varepsilon ,z} = \widehat{S}^{s}_{\varepsilon ,w}$ (green) is the projection of $\widehat{S}^{s}_{\varepsilon }$, which is identical in both projections because it lies on $\widetilde{\varSigma }_{1}$ so that $z=w$. An intersection between the two surfaces occurs when the three projected curves $\widehat{S}^{a}_{\varepsilon ,z}$, $\widehat{S}^{a}_{\varepsilon ,w}$ and $\widehat{S}^{s}_{\varepsilon ,z} = \widehat{S}^{s}_{\varepsilon ,w}$ all coincide in a single point. Hence, panels (c1) and (c2) show that there is no transversal intersection of $S^{a}_{\varepsilon }$ and $S^{s}_{\varepsilon }$, which represents the generic situation. On the other hand, there are many near intersections, which implies that there should be a number of orbit segments that lie on $S^{a}_{\varepsilon }$, stay extremely close to $S^{s}_{\varepsilon }$ for a finite time and then leave via $W^{u}(S^{s}_{\varepsilon })$. Such orbit segments are canard orbits as we discuss next.

### General Approach for Computing Canard Orbits in $\mathbb{R}^{4}$

In systems with one fast and two slow variables, canard orbits are defined as transversal intersections of slow manifolds [33, 34]. In systems with two fast and two slow variables, on the other hand, attracting and saddle slow manifolds are two dimensional and generically do not intersect, as was demonstrated in the previous section. This does not mean, however, that canard orbits do not exist in this higher-dimensional setting. Rather, canard orbits are trajectories that follow attracting and saddle slow manifolds for $\mathcal{O}(1)$ time on the slow time scale before exiting via the unstable manifold of the saddle slow manifold [65]. This well-known alternative definition of a canard orbit has the advantage that it does not depend on the dimension of the slow manifold. As such, we are able to propose a general approach for computing and continuing canard orbits as orbit segments that follow $S^{a}_{\varepsilon }$ and stay close to $S^{s}_{\varepsilon }$ for a significant amount of time before leaving via $W^{u}(S^{s}_{\varepsilon })$.

Consider system (1) and assume that the critical manifold has attracting and saddle sheets, $S^{a}$ and $S^{s}$, respectively, that meet at a generic fold curve *F* on which there is folded node. We compute canard orbits as orbit segments that satisfy the boundary conditions

29 $$ \textstyle\begin{cases} u(0) \in L^{a},\\ u(1) \in \widetilde{\varSigma }_{1} := \widehat{\varSigma }_{1} \cap \varSigma _{1}. \end{cases} $$

Here, $L^{a}$ is a one-dimensional curve on $S^{a}$ sufficiently far away from *F*. The section $\widetilde{\varSigma }_{1}$ is the two-dimensional intersection of three-dimensional submanifolds $\widehat{\varSigma }_{1}$ and $\varSigma _{1}$, where $\widehat{\varSigma }_{1}$ is chosen to be transverse to $W^{u}(S^{s}_{\varepsilon })$, and $\varSigma _{1}$ must be sufficiently far away from *F*. Note that $\widetilde{\varSigma }_{1}$ is chosen in the same way as for computing the saddle slow manifold; see (10). This computational setup forces the computed solution segments to follow $S^{a}_{\varepsilon }$ and then $S^{s}_{\varepsilon }$ for a sufficient amount of time before leaving via the unstable manifold $W^{u}(S^{s}_{\varepsilon })$. Such orbit segments provide excellent approximations of canard orbits, in the same spirit as those computed in [38].

To obtain a solution segment that satisfies (29), one needs to perform a homotopy step. The underlying idea is to start from an orbit segment with $u(0)\in L^{a}$ and $u(1)$ on either $\widehat{\varSigma }_{1}$ or $\varSigma _{1}$. We then continue this orbit segment until (29) is satisfied. Once detected, canard orbits that are solutions of the well-posed 2PBVP with the boundary conditions given by (29) can be continued in any system parameter.

#### Canard Orbits of the Extended Normal Form

#### Detecting and Continuing Canards of the Normal Form

To detect canard orbits of system (11), we represent them as orbit segments that satisfy (29) with the system-specific choice

30 $$ \textstyle\begin{cases} u(0) \in L^{a}_{1} = \{ z=-30 \} \cap S^{a}, \\ u(1) \in \widetilde{\varSigma }_{1} = \{ x=-z^{2} \} \cap \{ z=30 \} . \end{cases} $$

Note that $L^{a}_{1}$ is the same line as was used for computing the first and second submanifolds of $S^{a}_{\varepsilon }$, and $\widetilde{\varSigma }_{1} $ is the same section used for computing the first and second submanifolds of $S^{s}_{\varepsilon }$. Nonetheless, solutions of (30) do not correspond to transversal intersections of $S^{a}_{\varepsilon }$ and $S^{s}_{\varepsilon }$.

To detect a canard orbit $\xi _{i}$ with *i* small-amplitude oscillations (SAOs), we start from an orbit segment on $S^{a}_{\varepsilon }$ that exhibits *i* SAOs. We then extend this orbit segment by the flow until the end point reaches the section $\varSigma _{1} = \{ z=30 \} $. We then continue this orbit segment by imposing the boundary conditions

31 $$ \textstyle\begin{cases} u(0) \in L^{a}_{1} = \{ z=-30 \} \cap S^{a}, \\ u(1) \in \varSigma _{1} = \{ z=30 \} , \end{cases} $$

and letting $u(0)$ vary along $L^{a}_{1}$. This yields a one-parameter family of orbit segments on $S^{a}_{\varepsilon }$ with *i* SAOs. As soon as $u(1)$ lies on $\widehat{\varSigma }_{1} = \{ \dot{z}=0 \} $, Eq. (30) is satisfied and the corresponding orbit segment represents a canard orbit $\xi _{i}$.

**Fig. 7:**
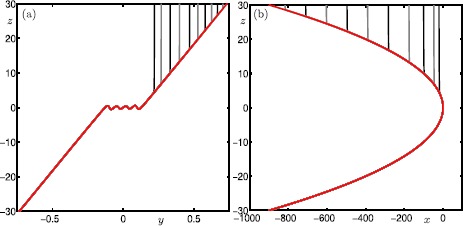
Homotopy step for detecting canard orbit $\xi _{4}$ of system (11) with $\mu =100.1$ and $\varepsilon =0.01$. Panels **(a)** and **(b)** are projections onto the ($y,z$)-plane and ($x,z$)-plane, respectively, and show a family of orbit segments of $S^{a}_{\varepsilon }$ that stay close to $S^{s}_{\varepsilon }$ for a certain amount of time. The red orbit, which stays close to $S^{s}_{\varepsilon }$ for the longest time, is identified as the canard orbit $\xi _{4}$ that satisfies (30)

Figure 7 illustrates this homotopy step for finding canard orbits in system (11) with $\mu =100.1$ and $\varepsilon =0.01$. Panels (a) and (b) show orbit segments with four SAOs that satisfy conditions (31), in projection onto the ($y,z$)-plane and ($x,z$)-plane, respectively. These orbits segments stay very close to $S^{s}_{\varepsilon }$ for a certain amount of time before leaving via $W^{u}(S^{s}_{\varepsilon })$. However, only the red orbit segment satisfies (30) and stays close to $S^{s}_{\varepsilon }$ for a longer time than all of the other computed orbit segments. This is because this orbit segment ‘switches’ to leave via $W^{u}(S^{s}_{\varepsilon })$ from the other side of the stable manifold $W^{s}(S^{s}_{\varepsilon })$. Hence, we regard it as the representative canard orbit $\xi _{4}$ with four SAOs. This homotopy step is an extension of the approach of detecting canard orbits in $\mathbb{R}^{3}$ that was presented in [38].

**Fig. 8:**
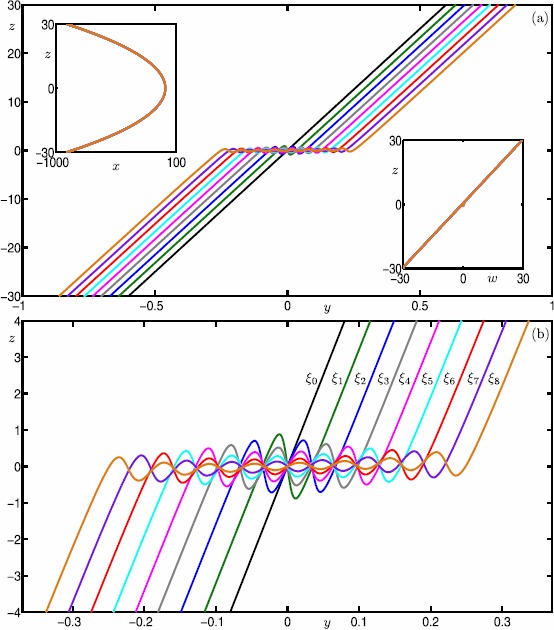
Canard orbits $\xi _{0}$–$\xi _{8}$ of system (11) with $\mu =100.1$ and $\varepsilon =0.01$. Panel **(a)** shows $\xi _{0}$–$\xi _{8}$ in projection onto the ($y,z$)-plane; and panel **(b)** is an enlargement. The insets show all computed canard orbits in projection onto the ($x,z$)-plane and ($w,z$)-plane, illustrating how close they are to the critical manifold *S*

For the parameter value $\mu =100.1$ and sufficiently small *ε*, the theory predicts the existence of two primary canards and 50 additional secondary canards [34]. Each of these canard orbits can be detected as solutions that satisfy (30), via the homotopy step discussed in Sect. 4.1.2. Figure 8 shows nine selected canard orbits $\xi _{0}$–$\xi _{8}$ of system (11) with $\mu =100.1$ and $\varepsilon =0.01$. Panel (a) shows the canard orbits in projection onto the ($y,z$)-plane, while the top left and bottom right insets show projections onto the ($x,z$)- and ($w,z$)-planes, respectively; panel (b) is an enlargement. The primary canard orbit $\xi _{0}$ follows both $S^{a}_{\varepsilon }$ and $S^{s}_{\varepsilon }$ without making any rotation. This canard orbit is the equivalent of the strong singular canard $\xi _{s}$ at the singular limit $\varepsilon =0$. Each canard orbit $\xi _{i}$, $i>0$, makes *i* rotations around the singular weak canard $\xi _{w}$ (not shown). Observe that the projections of canard orbits in the insets are close to those of the critical manifold *S*; see Eq. 4. This provides a strong evidence that all of the computed canard orbits $\xi _{0}$–$\xi _{8}$ stay extremely close to both $S^{a}$ and $S^{s}$. This is a clear indication that the computed orbit segments are accurate approximations of canard orbits.

#### Continuation of Canard Orbits

**Fig. 9:**
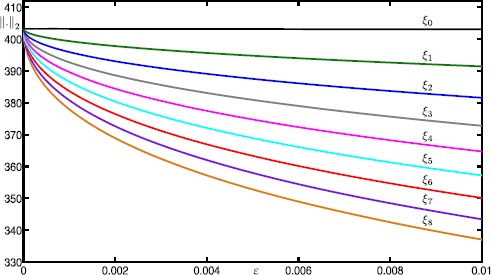
Continuation in *ε* of canard orbits $\xi _{0}$–$\xi _{8}$ for system (11) with $\mu = 100.1$

In this section, we perform an analysis of how the canard orbits depend on *μ* and *ε*. This is readily achieved by continuing them as orbit segments that satisfy the boundary conditions given by (30). Figure 9 shows the continuation of canard orbits $\xi _{0}$–$\xi _{8}$ in *ε*. We again use $\xi _{i}$ to denote the *ε*-dependent branches that correspond to canard orbits with *i* SAOs. Note that all branches converge to the singular limit $\varepsilon =0$. Indeed, we find that all canard orbits accumulate on the strong singular canard $\xi _{s}$ near the singular limit. This agrees with predictions by the theory [34].

**Fig. 10:**
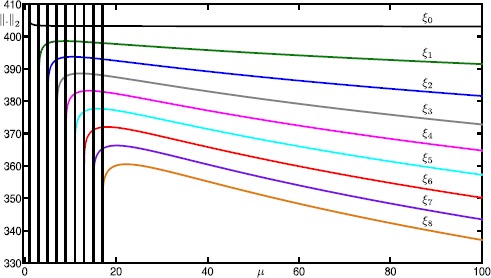
Continuation in *μ* of canard orbits $\xi _{0}$–$\xi _{8}$ for system (11) with $\varepsilon =0.01$

Figure 10 shows the continuation of canard orbits $\xi _{0}$–$\xi _{8}$ in *μ* for $\varepsilon =0.01$. The vertical lines are at the odd integer values of $\mu = 1,3,\ldots,17$. The figure illustrates how the branches $\xi _{0}$–$\xi _{8}$ terminate approximately at odd values of *μ*. More precisely, each branch $\xi _{i}$ ceases to exist at $\mu \approx 2i+1$ for all shown branches. According to the literature [34], the branches $\xi _{i}$ are expected to terminate exactly at $\mu = 2i+1$ near the singular limit $\varepsilon =0$. Again, our numerical results are consistent with the theory, which is another indication of the reliability of our computations.

## Slow Manifolds and Canard Orbits in the Full Hodgkin–Huxley Model

In this section, we implement and demonstrate our numerical approach for computing two-dimensional saddle slow manifolds and associated canard orbits in an example from neuroscience, namely, the Hodgkin–Huxley model. The Hodgkin–Huxley model was originally formulated to describe the action potential of the squid giant axon [8]. We study a singularly perturbed nondimensionalized version of the Hodgkin–Huxley model obtained by Rubin and Wechselberger [1], given by

32 $$ \textstyle\begin{cases} \dot{v} = f_{1}(v,m,h,n) := I/(k_{v} \cdot g)- m^{3} h (v- \bar{E}_{Na}) \\ \hphantom{\dot{v} = f_{1}(v,m,h,n) := }{}- \bar{g}_{k} n^{4} (v-\bar{E}_{K})- \bar{g}_{l} (v-\bar{E}_{L}), \\ \dot{m} = f_{2}(v,m) := \frac{1}{\tau _{m} t_{m}(v)} (m_{\infty }(v)- m), \\ \dot{h} = \varepsilon g_{1}(v,h) := \frac{\varepsilon }{\tau _{h} t_{h}(v)} (h_{\infty }(v)- h), \\ \dot{n} = \varepsilon g_{2}(v,n) := \frac{\varepsilon }{\tau _{n} t_{n}(v)} (n_{\infty }(v)- n), \end{cases} $$

where *v* represents the action potential and $m, h$ and *n* represent gating variables. The parameter $I>0$ is the injected current and *ε* is the time-scale separation parameter. The steady-state functions $m_{\infty }(v)$, $h_{\infty }(v)$ and $n_{\infty }(v)$, and time functions $t_{m}(v)$, $t_{h}(v)$ and $t_{m}(v)$ are standard from the Hodgkin–Huxley formalism, and they are given in the Appendix. In this paper, we vary *I* and *ε* and keep the other parameters fixed as in Table 1; the given parameter values are the same used in [1] and they correspond to the original Hodgkin–Huxley model, except that we set $\tau _{h} = 2$ instead of $\tau _{h} = 1$ to obtain more interesting dynamics. We initially set $I=9.74$ and $\varepsilon =0.0083$. For *ε* small, *v* and *m* can be regarded as fast variables, and *h* and *n* as slow variables.

**Table 1:** Parameter values of (32)

$\tau _{m}$	$\tau _{h}$	$\tau _{n}$	$E_{Na}$	$E_{K}$	$E_{L}$	*g*	$g_{k}$	$g_{l}$	$k_{v}$
1	2	1	0.5	−0.77	−0.544	120	0.3	0.0025	100

Rubin and Wechselberger [1] applied a center manifold reduction to (32) by setting $m=m_{\infty }(v)$ to eliminate *m* and obtain a three-dimensional reduced model. They applied methods from GSPT to prove the existence of relaxation oscillations, MMOs and canard orbits in the three-dimensional reduced model. They also performed bifurcation analyses in *I* and *ε* for various choices of $\tau _{h}$ and $\tau _{n}$ [42]. Desroches et al. [36] detected and continued canard orbits for the same reduced three-dimensional model. Other versions of the full four-dimensional Hodgkin–Huxley model have been investigated in previous studies [8, 66–69]. Here, we study the nondimensionalized version of the full Hodgkin–Huxley model (32) without applying any reduction. More specifically, we examine the role of slow manifolds and associated canard orbits in organizing MMOs.

### Bifurcation Diagram of the Four-Dimensional Hodgkin–Huxley Model

**Fig. 11:**
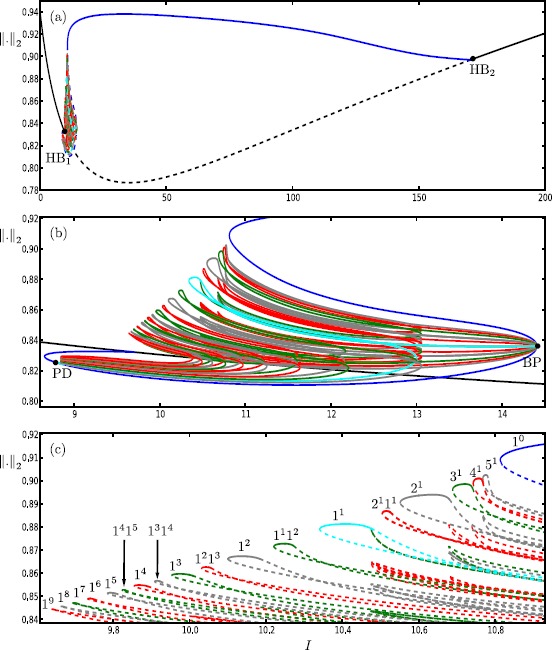
One-parameter bifurcation diagram of the Hodgkin–Huxley model (32) showing the $L_{2}$-norm versus *I*. The black curve is the branch of equilibria and colored curves are branches of periodic orbits; the curves are solid when stable and dashed when unstable. Panel **(b)** is an enlargement near HB_1_ that shows more branches of periodic orbits; stability is not shown here. Panel **(c)** is a further enlargement that shows the stability and the MMO signatures of the branches of periodic orbits

We start our analysis with the bifurcation diagram of the Hodgkin–Huxley model (32) in the parameter *I* for fixed $\varepsilon =0.0083$. Figure 11 shows the $L_{2}$-norm of equilibria (black curve) and periodic orbits (colored curves) versus *I*; solid and dashed curves indicate stable and unstable branches, respectively. Note that there is a single equilibrium point for $I \in [0,200]$. It undergoes subcritical and supercritical Hopf bifurcations (black dots) at $I \approx 9.70009$ and $I \approx 170.609$ (labeled HB_1_ and HB_2_), respectively. In panel (a), the primary branch of periodic orbits (blue curve) connects HB_1_ with HB_2_. Panel (b) shows an enlargement for $I \in [8.5986,14.5045]$; stability properties are not shown here. The primary branch undergoes a period-doubling bifurcation (PD) at $I \approx 8.80514$. The period-doubled branch (cyan) that emanates from PD terminates at a branch point (BP) at $I \approx 14.4103$. Figure 11(b) also shows 17 other branches of periodic orbits that are alternately colored gray, red and green; these branches are isolas and some of them have folds extremely close to the point BP. Panel (c) shows an enlargement of panel (b) with stability properties shown. Each branch goes through a number of saddle-node bifurcations of periodic orbits and period-doubling bifurcations. Therefore, different types of stabilities can be found along these branches. The labels indicate the MMO signatures of the stable parts at the top of the branches. For increasing values of *I*, the number of large-amplitude oscillations (LAOs) increases and the number of small-amplitude oscillations (SAOs) decreases. Note that the branches 1^1^2^1^, 1^1^1^2^, 1^2^1^3^, 1^3^1^4^ and 1^4^1^5^ are combinations of signatures of the neighboring branches [19, 70]. The periodic orbits that lie on a given branch are topologically equivalent; however, the oscillations exhibited by the periodic orbits vary in amplitude along that branch. For example, the branch labeled 5^1^ also includes periodic orbits with signatures of type ${L_{1}}^{s_{1}} {L_{2}}^{s_{2}}\ldots$ , where $\sum_{i=1} L_{i}+s_{i}=6$. It is notable that some branches coexist for a range of parameter values. For instance, we find at least 26 coexisting periodic orbits for $I=9.74$. For the remainder of this paper, we fix $I=9.74$ and investigate the structure of the slow manifolds and other dynamics of (32).

### Slow–Fast Analysis of the Four-Dimensional Hodgkin–Huxley Model

**Fig. 12:**
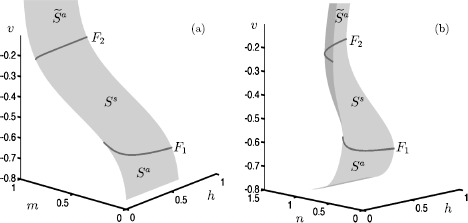
Critical manifold of the Hodgkin–Huxley model (32) with $I=9.74$, projected onto the $(m,h,v)$-space **(a)** and $(n,h,v)$-space **(b)**

To gain insight into the geometric mechanism of MMOs, we start with applying methods from GSPT to the four-dimensional Hodgkin–Huxley model. Taking the singular limit $\varepsilon = 0$ of (32) yields the two-dimensional fast subsystem

33 $$ \textstyle\begin{cases} \dot{v} = f_{1}(v,m,h,n), \\ \dot{m} = f_{2}(v,m), \\ \dot{h} = 0, \\ \dot{n} = 0, \end{cases} $$

where *h* and *n* can be treated as bifurcation parameters. Equilibrium points of (33) form a two-dimensional surface corresponding to the critical manifold *S*, which is a cubic-shaped smooth surface with two fold curves. Figure 12 shows the critical manifold for $I=9.74$ in two different projections. The two fold curves $F_{1}$ and $F_{2}$ are generic fold (saddle-node) bifurcations of (33), and they separate the critical manifold into three different sheets. Note that the values of *v* and *m* hardly change along the upper fold curve $F_{2}$. The lower and upper sheets ($S^{a}$, $\widetilde{S}^{a}$) of the critical manifold are attracting since the corresponding two normal eigenvalues of the equilibria are negative. The middle sheet $S^{s}$ is of saddle type since the corresponding equilibria have eigenvalues of opposite signs.

The reduced system of (32) is the two-dimensional system of differential algebraic equations:

34 $$ \textstyle\begin{cases} 0 = f_{1}(v,m,h,n), \\ 0 = f_{2}(v,m), \\ h^{\prime } = g_{1}(v,h), \\ n^{\prime } = g_{2}(v,n), \end{cases} $$

where the prime denotes the derivative with respect to the slow time scale $\tau = \varepsilon t$. With the implicit function theorem, the reduced system can be written in the form

35 $$ \textstyle\begin{cases} - ( \frac{\partial f_{1}}{\partial v} - \frac{\partial f_{1}}{\partial m} \cdot (\frac{\partial f_{2}}{\partial v} / \frac{\partial f_{2}}{\partial m}) ) v^{\prime } = \frac{\partial f_{1}}{\partial h} g_{1}(v,h)+ \frac{\partial f_{1}}{\partial n} g_{2}(v,n), \\ h^{\prime } = g_{1}(v,h). \end{cases} $$

Straightforward calculations show that system (35) is singular along the fold curves $F_{1}$ and $F_{2}$. This problem can be avoided via time rescaling by the factor $-(\frac{\partial f_{1}}{\partial v} - \frac{\partial f_{1}}{\partial m} \cdot (\frac{\partial f_{2}}{\partial v} / \frac{\partial f_{2}}{\partial m}))$ to obtain

36 $$ \textstyle\begin{cases} v^{\prime } = \frac{\partial f_{1}}{\partial h} g_{1}(v,h)+ \frac{\partial f_{1}}{\partial n} g_{2}(v,n), \\ h^{\prime } = - ( \frac{\partial f_{1}}{\partial v} - \frac{\partial f_{1}}{\partial m} \cdot (\frac{\partial f_{2}}{\partial v} / \frac{\partial f_{2}}{\partial m}) ) g_{1}(v,h), \end{cases} $$

where the prime now denotes the derivative with respect to the new rescaled time

37 $$ \tau _{1} = - \tau / \biggl( \frac{\partial f_{1}}{\partial v} - \frac{\partial f_{1}}{\partial m} \cdot \biggl(\frac{\partial f_{2}}{\partial v} / \frac{\partial f_{2}}{\partial m} \biggr) \biggr) . $$

System (36) is the desingularized reduced system, for which the orientation on the saddle sheet $S^{s}$ of the critical manifold is reversed. We found that, for $I=9.74$, the desingularized reduced system (36) features two equilibrium points. One equilibrium lies on the saddle sheet $S^{s}$ and corresponds to a saddle-focus equilibrium point *q* of the full system (32), given by

38 $$ q = (v,m,h,n) \approx (-0.596423, 0.973836, 0.405820, 0.401974). $$

This equilibrium point has a two-dimensional stable manifold $W^{s}(q)$ and a two-dimensional unstable manifold $W^{u}(q)$. The other equilibrium of (36) lies on the lower fold curve $F_{1}$ and corresponds to the folded node singularity:

39 $$ p = (v,m,h,n) \approx (-0.599361,0.094301,0.334475,0.395965). $$

The strong and weak stable manifolds of *p* in system (36) correspond to the strong singular canard $\xi _{s}$ and the weak singular canard $\xi _{w}$. This allows for the existence of the two-dimensional funnel region, on which solutions of system (35) move from the lower attracting sheet $S^{a}$ through *p* to the saddle sheet $S^{s}$ in finite time; compare with Fig. 1. The eigenvalues of *p* are approximately −0.003945 and −0.000125 and their ratio is 31.56. Based on this ratio, the theory [34] predicts that the folded node gives rise to two primary canard orbits and 15 secondary canard orbit near the singular limit $\varepsilon =0$.

Recall that, for *ε* sufficiently small, the sheets $S^{a}$ and $S^{s}$ perturb to attracting and saddle slow manifolds $S^{a}_{\varepsilon }$ and $S^{s}_{\varepsilon }$, respectively. Additionally, the stable $W^{s}(S^{s})$ and unstable manifolds $W^{u}(S^{s})$ of the saddle sheet $S^{s}$ also persist as stable $W^{s}(S^{s}_{\varepsilon })$ and unstable $W^{u}(S^{s}_{\varepsilon })$ fast manifolds of the saddle slow manifold $S^{s}_{\varepsilon }$. We aim to use the numerical techniques introduced in Sect. 3 to compute slow manifolds and associated canard orbits, and subsequently examine the local and global influence of their geometry on the dynamics of the Hodgkin–Huxley model (32).

### Computing the Attracting Slow Manifold

**Fig. 13:**
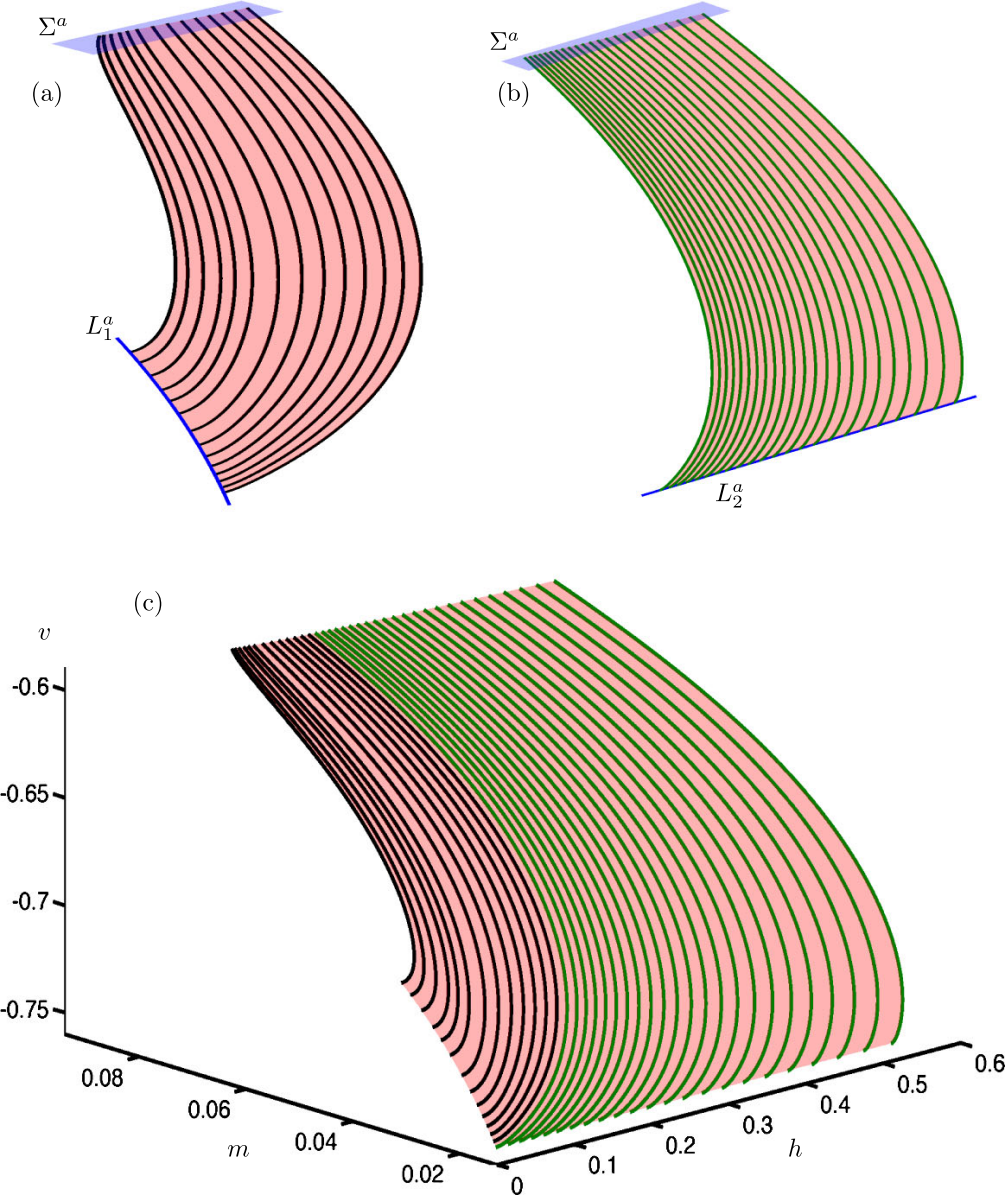
Computation of the lower attracting slow manifold $S^{a}_{\varepsilon }$ of the Hodgkin–Huxley model (32) with $\varepsilon =0.0083$ and $I=9.74$. Panels **(a)** and **(b)** show two submanifolds of $S^{a}_{\varepsilon }$ (red surfaces) together with their respective boundary conditions $L^{a}_{1}$ and $L^{a}_{2}$ (blue lines), and $\varSigma ^{a}$ (blue planes); they are rendered from the orbit segments shown as thick curves (black and green, respectively). Panel **(c)** shows both submanifolds of $S^{a}_{\varepsilon }$ together as a single surface

In order to understand the underlying dynamics of the Hodgkin–Huxley model (32), we perform computations of the locally invariant slow manifolds. The attracting slow manifold $S^{a}_{\varepsilon }$ of system (32) can be computed with the generalized setup for computing attracting and repelling slow manifolds that was introduced in Sect. 3.2. More specifically, we represent submanifolds of $S^{a}_{\varepsilon }$ as families of orbit segments that satisfy the 2PBVP setup given by (8), where $L^{a}$ is a line on the lower attracting sheet $S^{a}$ sufficiently far away from the lower fold curve $F_{1}$, and $\varSigma ^{a}$ is a suitably chosen three-dimensional submanifold transverse to the attracting slow manifold. Figure 13 shows two computed submanifolds of the attracting slow manifold $S^{a}_{\varepsilon }$ associated with the lower attracting sheet $S^{a}$. Panel (a) shows the first submanifold of $S^{a}_{\varepsilon }$ (red surface), which is rendered from a family of orbit segments (black) that satisfy the system-specific boundary conditions

40 $$ \textstyle\begin{cases} u(0) \in L^{a}_{1} := \{ (v,m,h,n) \mid h=0.1 \} \cap S^{a}, \\ u(1) \in \varSigma ^{a} := \{ (v,m,h,n) \mid v=-0.6 \} . \end{cases} $$

Figure 13(b) shows the second submanifold of $S^{a}_{\varepsilon }$ (red surface), which is rendered with a different family of orbit segments (green), satisfying the boundary conditions

41 $$ \textstyle\begin{cases} u(0) \in L^{a}_{2} := \{ (v,m,h,n) \mid v=-0.754 \} \cap S^{a}, \\ u(1) \in \varSigma ^{a} := \{ (v,m,h,n) \mid v=-0.6 \} . \end{cases} $$

Panel (c) shows the overall attracting slow manifold $S^{a}_{\varepsilon }$ near *p* consisting of the two computed families of orbit segments shown in panels (a) and (b).

### Computing the Saddle Slow Manifold

**Fig. 14:**
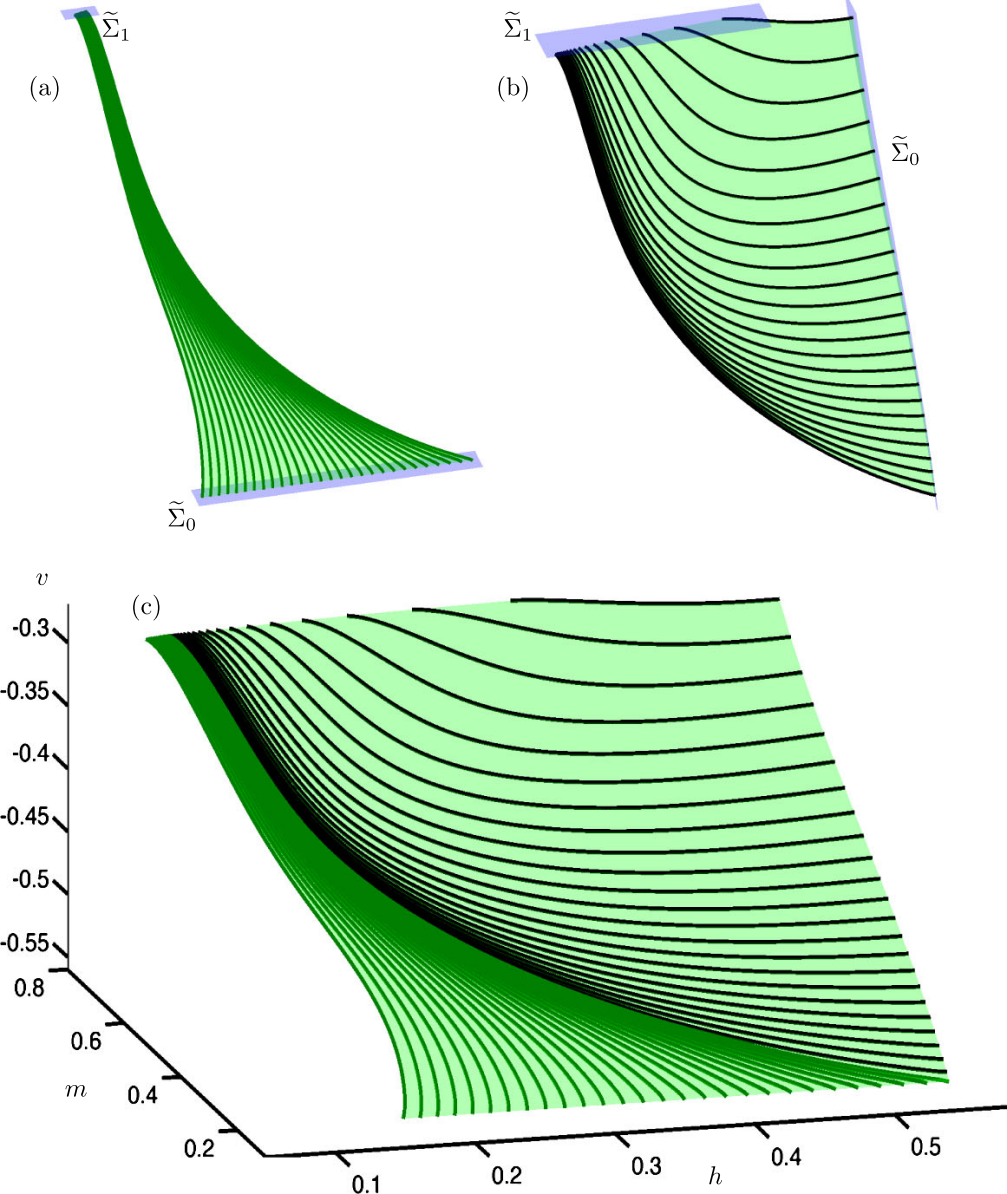
Computation of the saddle slow manifold $S^{s}_{\varepsilon }$ of the Hodgkin–Huxley model (32) with $\varepsilon =0.0083$ and $I=9.74$. Panels **(a)** and **(b)** show two submanifolds of $S^{s}_{\varepsilon }$ (green surfaces) together with their respective boundary conditions $\widetilde{\varSigma }_{0}$ and $\widetilde{\varSigma }_{1}$ (blue planes); they are rendered from the orbit segments shown as thick curves (black and green, respectively). Panel **(c)** shows both submanifolds of $S^{s}_{\varepsilon }$ together as a single surface

We now implement the approach introduced in Sect. 3.2 to compute the two-dimensional saddle slow manifold $S^{s}_{\varepsilon }$ of (32) by representing it as a family of orbit segments computed with the 2PBVP setup given by (9) and (10). As for $S^{a}_{\varepsilon }$, we represent the saddle slow manifold $S^{s}_{\varepsilon }$ with two submanifolds, which are shown in Fig. 14. Panel (a) shows a green surface, which is rendered from the first family of orbit segments (green). Panel (b) shows the second submanifold of $S^{s}_{\varepsilon }$ (green surface) rendered from a different family of orbit segments (black). In panels (a) and (b), the sections $\widetilde{\varSigma }_{0}$ and $\widetilde{\varSigma }_{1}$ (blue planes) correspond to the boundary conditions of the respective computed orbit segments. These sections are defined by (10), where the three-dimensional submanifolds $\widehat{\varSigma }_{0}$, $\widehat{\varSigma }_{1}$, $\varSigma _{0}$ and $\varSigma _{1}$ are chosen as follows. The sections $\widehat{\varSigma }_{0}$ and $\widehat{\varSigma }_{1}$ of (10) must be transverse to the associated stable manifold $W^{s}(S^{s}_{\varepsilon })$ and unstable manifold $W^{u}(S^{s}_{\varepsilon })$, respectively. We use a different section $\widehat{\varSigma }_{0}$ for each submanifold of the computed saddle slow manifold to ensure that the corresponding orbit segments are transverse to $W^{s}(S^{s}_{\varepsilon })$. Specifically, we choose

42 $$ \widehat{\varSigma }_{0} := \bigl\{ (v,m,h,n) \mid \dot{m} =0 \bigr\} = \bigl\{ (v,m,h,n) \mid f_{2}(v,m) =0 \bigr\} $$

for the black orbit segments, and

43 $$ \widehat{\varSigma }_{0} := \bigl\{ (v,m,h,n) \mid \dot{v} =0 \bigr\} = \bigl\{ (v,m,h,n) \mid f_{1}(v,m,h,n) =0 \bigr\} $$

for the green orbit segments. The section

44 $$ \widehat{\varSigma }_{1} := \bigl\{ (v,m,h,n) \mid \dot{v} =0 \bigr\} = \bigl\{ (v,m,h,n) \mid f_{1}(v,m,h,n) =0 \bigr\} $$

is used for both submanifolds of $S^{s}_{\varepsilon }$, and it is chosen transverse to $W^{u}(S^{s}_{\varepsilon })$. In contrast, sections $\varSigma _{0}$ and $\varSigma _{1}$ determine the constraints of the computed submanifold of $S^{s}_{\varepsilon }$. More specifically, for the green orbit segments, we choose

45 $$ \textstyle\begin{cases} \varSigma _{0} := \{ (v,m,h,n) \mid v=-0.55 \} , \\ \varSigma _{1} := \{ (v,m,h,n) \mid v=-0.28 \} , \end{cases} $$

so that these orbit segments start near the fold curve $F_{1}$ and end at $v=-0.28$. For the black orbit segments, we choose

46 $$ \textstyle\begin{cases} \varSigma _{0} := \{ (x,y,z,w) \mid h=0.55 \} , \\ \varSigma _{1} := \{ (x,y,z,w) \mid v=-0.28 \} , \end{cases} $$

which means that these orbit segments start from $h=0.55$ instead. Figure 14(c) shows both submanifolds together, so that they form the entire surface, that is, the saddle slow manifold $S^{s}_{\varepsilon }$.

### Interaction Between Attracting and Saddle Slow Manifolds

**Fig. 15:**
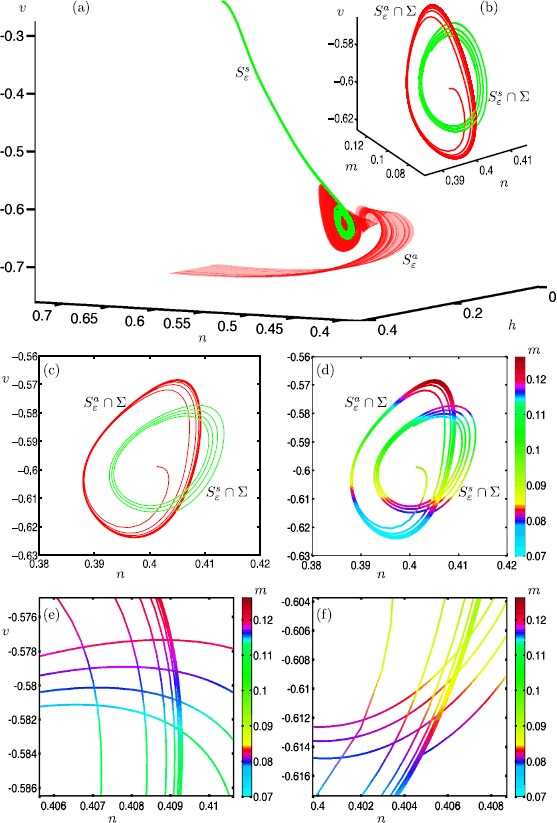
Interaction between the slow manifolds $S^{a}_{\varepsilon }$ and $S^{s}_{\varepsilon }$ of system (32) with $\varepsilon =0.0083$ and $I=9.74$. Panel **(a)** shows $S^{a}_{\varepsilon }$ (red surface) and $S^{s}_{\varepsilon }$ (green surface) computed up the section *Σ*. Panel **(b)** shows the intersection curves $S^{a}_{\varepsilon } \cap \varSigma $ (red) and $S^{s}_{\varepsilon } \cap \varSigma $ (green) in the ($m,n,v$)-space representing *Σ*; panel **(c)** is a projection onto the ($n,v$)-plane. Panel **(d)** shows intersection curves $S^{a}_{\varepsilon } \cap \varSigma $ and $S^{s}_{\varepsilon } \cap \varSigma $ in projection onto the ($n,v$)-plane, where the value of *m* is color coded as shown by the color bar; panels **(e)** and **(f)** are enlargements

Attracting and saddle slow manifolds of system (32) can be extended by the flow in forward and backward time, respectively, to study the SAOs that occur in the vicinity of the lower fold curve $F_{1}$. The mechanism for these SAOs can be explained by the combined presence of the folded node *p* given by (39) and the saddle-focus equilibrium *q* given by (38). Figure 15 shows the computations of the attracting slow manifold $S^{a}_{\varepsilon }$ and the saddle slow manifold $S^{s}_{\varepsilon }$ extended up to the three-dimensional cross section $\varSigma := \{ h=0.4 \} $. Panel (a) shows $S^{a}_{\varepsilon }$ (red) and $S^{s}_{\varepsilon }$ (green) in projection onto ($n,h,v$)-space. Panel (b) shows the intersection curves $S^{a}_{\varepsilon } \cap \varSigma $ (red) and $S^{s}_{\varepsilon } \cap \varSigma $ (green) in the ($m,n,v$)-space representing *Σ*. Since they are again very close to each other, it is very challenging to know whether there are any intersections between the two curves. Therefore, we use a different projection technique to gain a better insight. Panel (c) shows the intersection curves $S^{a}_{\varepsilon } \cap \varSigma $ and $S^{s}_{\varepsilon } \cap \varSigma $, projected onto the ($n,v$)-plane. Panel (d) illustrates the same intersection curves with the values of *m* color coded as shown by the color bar; panels (e) and (f) are enlargements of panel (d). We find no intersections between the curves $S^{a}_{\varepsilon } \cap \varSigma $ and $S^{s}_{\varepsilon } \cap \varSigma $, as is expected for two curves in $\mathbb{R}^{4}$. Nevertheless, the colors show that there are again many near intersections. Hence, as before, there should be orbit segments on $S^{a}_{\varepsilon }$ that stay extremely close to the saddle slow manifold $S^{s}_{\varepsilon }$ before leaving via its unstable manifold $W^{u}(S^{s}_{\varepsilon })$. Such orbit segments are canard orbits in $\mathbb{R}^{4}$, as was discussed Sect. 4.

### Computing Canard Orbits of the Hodgkin–Huxley Model

We now implement the general approach introduced in Sect. 4.1 to detect canard orbits of system (32). More specifically, we compute them as orbit segments that lie on the attracting slow manifold $S^{a}_{\varepsilon }$ and stay extremely close to the saddle slow manifold $S^{s}_{\varepsilon }$ before leaving via its unstable manifold $W^{u}(S^{s}_{\varepsilon })$; see equations (29).

Here, we use a different homotopy step from the one used for system (11). To detect a canard orbit $\xi _{i}$ with *i* SAOs, we start from a solution that lies on $S^{a}_{\varepsilon }$ and exhibits *i* SAOs in the vicinity of the lower fold $F_{1}$. We then continue this solution as a one-parameter family of orbit segments subject to the boundary conditions

47 $$ \textstyle\begin{cases} u(0) \in L_{2}^{a}, \\ u(1) \in \widehat{\varSigma }_{1} . \end{cases} $$

Here, $L_{2}^{a}$ is the line defined in (41) that we used for computing the second submanifold of $S^{a}_{\varepsilon }$, and $\widehat{\varSigma }_{1} $ is the section defined by (44). These boundary conditions (47) yield a family of orbit segments on $S^{a}_{\varepsilon }$ that terminate at the *v*-nullcline, which is transverse to the unstable manifold $W^{u}(S^{s}_{\varepsilon })$. They also guarantee that the orbit segments exhibit exactly *i* SAOs.

**Fig. 16:**
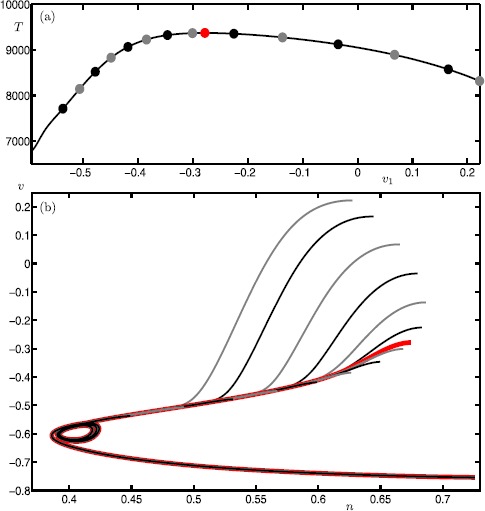
Approach for detecting canard orbits in the Hodgkin–Huxley model (32) with $\varepsilon =0.0083$ and $I=9.74$. Panel **(a)** shows the integration time *T* versus the *v*-values $v_{1}$ of the end points of orbit segments that satisfy (47); the black, gray and red dots correspond to a selection of the computed orbits segments. Panel **(b)** shows these selected orbit segments from panel **(a)** in their respective colors, in projection onto the ($n,v$)-plane

Figure 16 shows this homotopy step for computing canard orbits for system (32). Panel (a) illustrates how the integration time *T* depends on the *v*-coordinate $v_{1}$ of the end points on $\widehat{\varSigma }_{1}$ for a family of orbit segments that satisfy (47); the black, gray and red dots correspond to a selection of the computed orbits segments. Panel (b) shows the orbit segments that correspond to the dots of panel (a) in their respective colors, in projection onto the $(n,v)$-plane. The shown orbit segments start from a very small interval on $L_{2}^{a}$. More specifically, their initial *h*-values along $L_{2}^{a}$ agree to 6 decimal places, namely, $h \approx 0.135841$. All shown orbits follow $S^{a}_{\varepsilon }$, exhibit four SAOs, and then follow $S^{s}_{\varepsilon }$ for a certain amount of time before leaving via $W^{u}(S^{s}_{\varepsilon })$. Thus, these orbit segments are indeed transversal intersections of $S^{a}_{\varepsilon }$ and $W^{u}(S^{s}_{\varepsilon })$. The thick red orbit segment corresponds to the trajectory that stays close to $S^{s}_{\varepsilon }$ for the longest time, and as such, we regard it as the representative canard orbit $\xi _{4}$.

We performed the same homotopy step to detect other canard orbits $\xi _{i}$ with *i* SAOs in system (32) and found that, for all *i*, the orbit segment that spends the longest time near $S^{s}_{\varepsilon }$ has a *v*-maximum of $v \approx -0.28$ rounded to the second decimal place. Therefore, we compute all canard orbits as orbit segments that satisfy (47) and have a *v*-maximum of $v = -0.28$. More precisely, we compute all canard orbits of system (32) as orbit segments that satisfy the boundary conditions

48 $$ \textstyle\begin{cases} u(0) \in L_{2}^{a}, \\ u(1) \in \widetilde{\varSigma }_{1} = \widehat{\varSigma }_{1} \cap \varSigma _{1}. \end{cases} $$

Note that $\widetilde{\varSigma }_{1}$ is the section we used for computing both submanifolds of $S^{s}_{\varepsilon }$; see equations (44), (45) and (46).

#### Selection of Canard Orbits for $I=9.74$

**Fig. 17:**
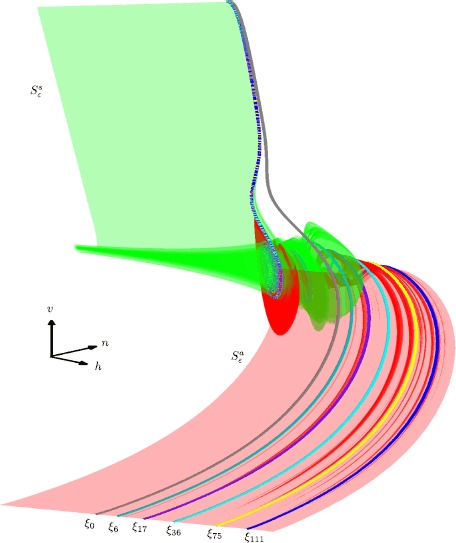
Selected canard orbits (thick curves) plotted together with $S^{a}_{\varepsilon }$ (red) and $S^{s}_{\varepsilon }$ (green), in projection onto the ($v,h,n$)-space, of the Hodgkin–Huxley model (32) with $\varepsilon =0.0083$ and $I=9.74$

Figure 17 shows six selected canard orbits (thick curves) together with the extended attracting and saddle slow manifolds. The slow manifolds $S^{a}_{\varepsilon }$ (red surface) and $S^{s}_{\varepsilon }$ (green surface) are extended by the flow to include the SAOs in the vicinity of the lower fold curve $F_{1}$. The primary canard orbit $\xi _{0}$ separates trajectories on ${S^{a}_{\varepsilon }}$ that make at least one rotation around *F* from those that escape the fold region without making any rotations. Each canard orbit $\xi _{i}$, $i>0$, makes *i* SAOs in the vicinity of the fold. Recall that there should exist 15 secondary canard orbits for $I=9.74$ near the singular limit $\varepsilon =0$. Nevertheless, we detect more than 111 canard orbits for $\varepsilon =0.0083$, and $\xi _{111}$ is shown as part of the selection in Fig. 17.

### Ribbons of the Attracting Slow Manifolds

In [38], we showed that the signature of a given MMO in the three-dimensional autocatalator introduced in [71] is directly related to the nearby canard orbits and so-called ribbons of the attracting slow manifolds. Such ribbons are separate surfaces, bounded by pairs of canard orbits that exhibit the same number of SAOs. We use the term twin canard orbits to describe a pair of canard orbits that bound a ribbon. We find a very similar structure of ribbons and associated twin canard orbits in the four-dimensional Hodgkin–Huxley model (32).

**Fig. 18:**
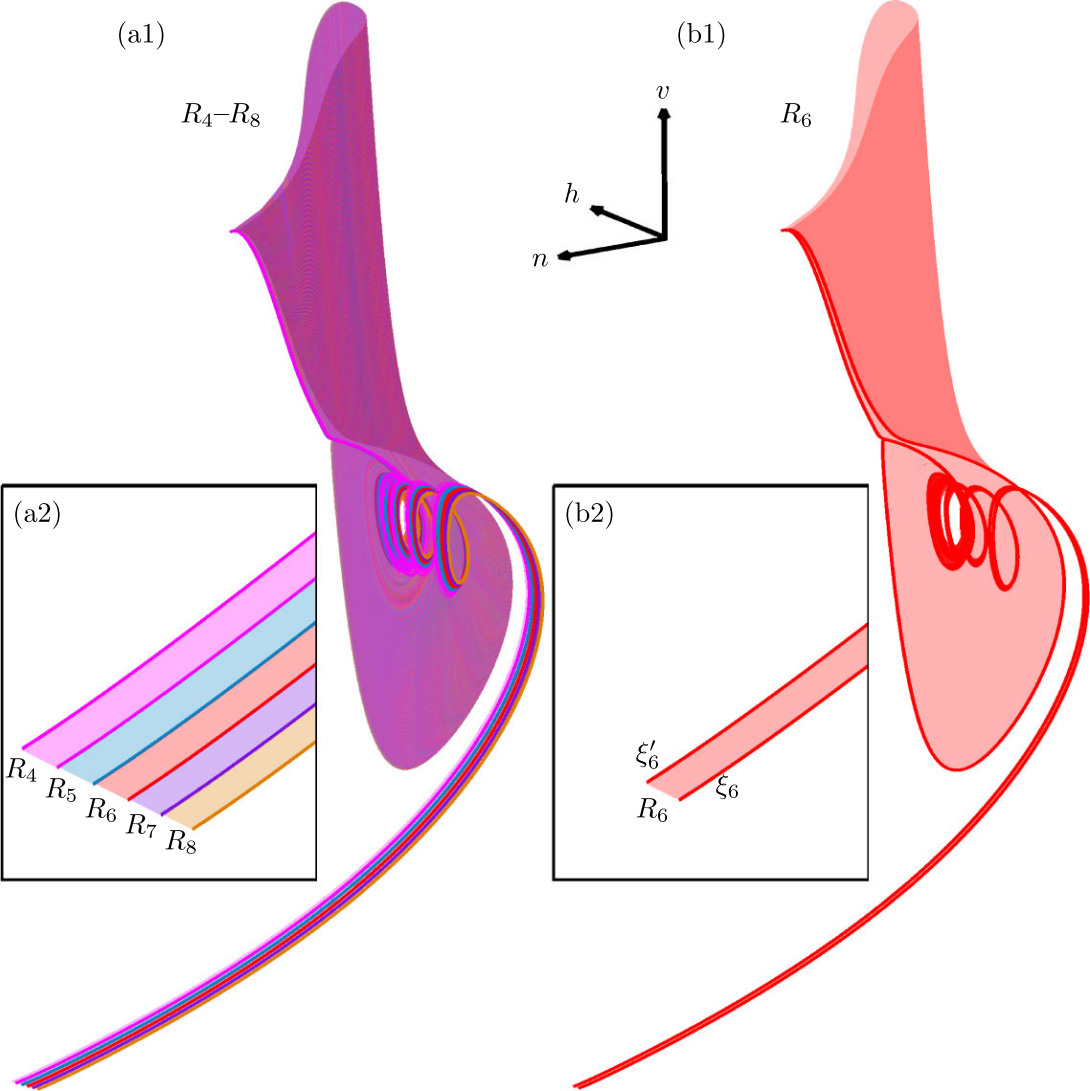
Ribbons of the extended attracting slow manifold in the Hodgkin–Huxley model (32) with $\varepsilon =0.0083$ and $I=9.74$. Panel **(a)** shows five ribbons $R_{4}$–$R_{8}$ of the extended attracting slow manifold in five different colors, together with the associated canard orbits (thick curves); the inset is an enlargement. Panel **(b)** shows the ribbon $R_{6}$ with the bounding twin canard orbits $\xi _{6}$ and $\xi '_{6}$ (thick curves); the inset is again an enlargement

Similar to the approach taken in [38], we extend $S^{a}_{\varepsilon }$ by the flow up to the three-dimensional cross section $\varSigma _{1}$, which was chosen as part of the selected boundary conditions for computing $S^{s}_{\varepsilon }$ and associated canard orbits; see equations (45), (46), and (48). Figure 18 illustrates a selection of ribbons of the attracting slow manifold $S^{a}_{\varepsilon }$ for system (32). Panel (a) shows five ribbons $R_{4}$–$R_{8}$ of the extended attracting slow manifold $S^{a}_{\varepsilon }$ in five different colors. The inset (a2) is an enlargement near the line $L_{2}^{a}$ where the ribbons are easily distinguished. Each ribbon $R_{i}$ is computed individually as a family of orbit segments that start from $L_{2}^{a}$, make *i* SAOs, and end at the cross section $\varSigma _{1}$. Figure 18(b) shows ribbon $R_{6}$ (red surface) and the bounding canard orbits $\xi _{6}$ and $\xi '_{6}$ (thick red curves). The inset (b2) is an enlargement near $L_{2}^{a}$. This ribbon $R_{6}$ is representative of the other ribbons $R_{i}$, each of which is bounded by a pair of canard orbits $\xi _{i}$ and $\xi '_{i}$ that exhibit *i* SAOs. We again refer to the canard orbits $\xi _{i}$ and $\xi '_{i}$ as twin canard orbits. Moreover, each ribbon $R_{i}$ rotates as a surface around the fold curve $F_{1}$ and makes *i* SAOs before reaching $\varSigma _{1}$. Notice also from Fig. 18 that each ribbon $R_{i}$ folds over sharply in the process.

**Fig. 19:**
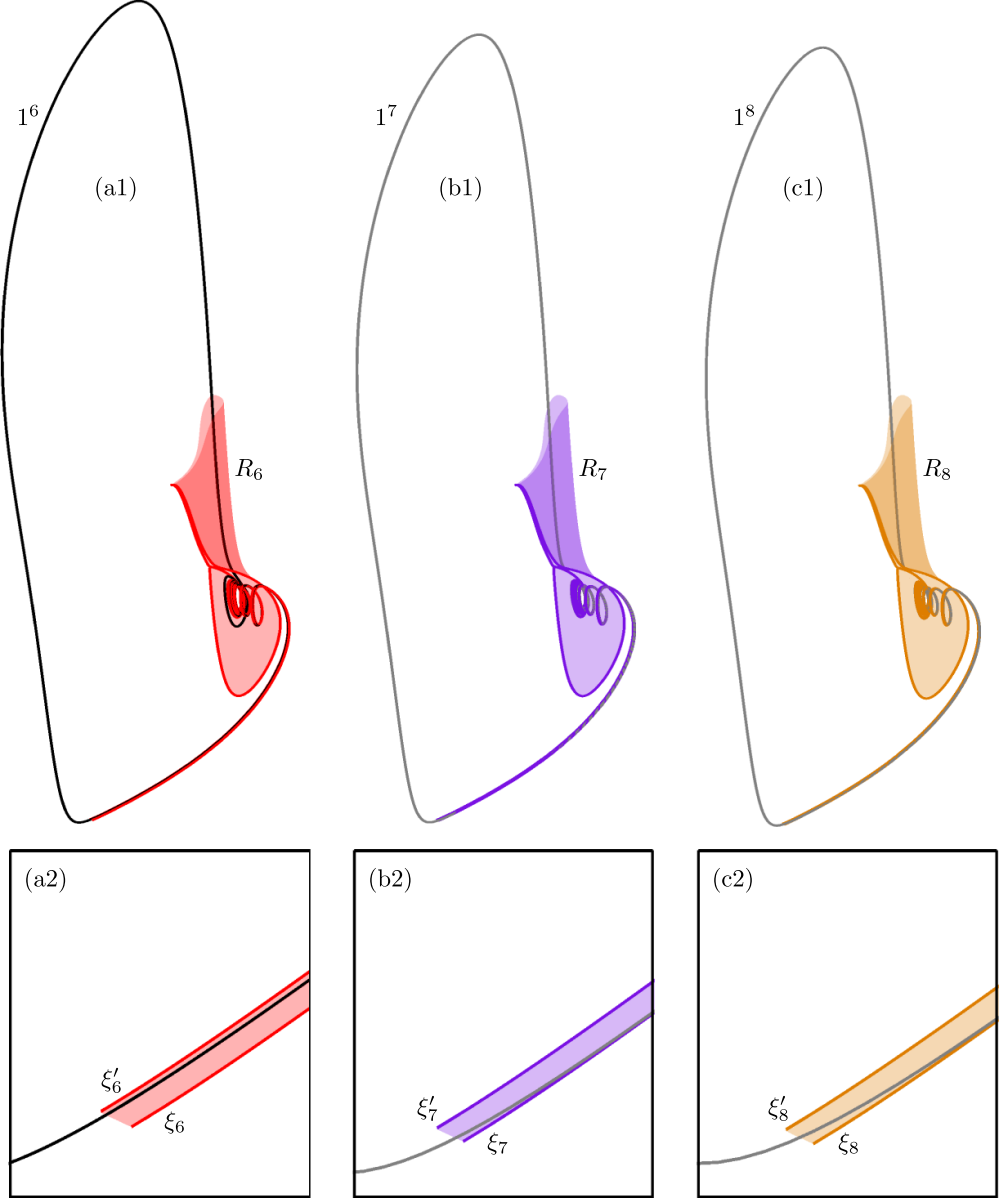
Periodic MMOs with signatures 1^6^–1^8^ of the Hodgkin–Huxley model (32) with $\varepsilon =0.0083$ and $I=9.74$, plotted together with their associated ribbons $R_{6}$–$R_{8}$ of the extended attracting slow manifold. Panels **(a)**–**(c)** show the stable MMO 1^6^ (black) and the unstable MMOs 1^7^ and 1^8^ (gray), respectively. The insets are enlargements near the line $L^{a}_{2}$. Each ribbon $R_{i}$ is bounded by its respective pair of twin canard orbits $\xi _{i}$ and $\xi '_{i}$

As mentioned before in Sect. 5.1, we find more than 26 periodic orbits for $I=9.74$ with different MMO signatures. We find that ribbons of the extended attracting slow manifold and associated twin canard orbits give great insight into the mechanism of these different MMO signatures. Figure 19 shows three different periodic MMOs with different signatures superimposed on the associated ribbons of the extended attracting slow manifold for (32) with $I=9.74$. Panels (a), (b) and (c) show the stable periodic MMO with signature 1^6^ (black orbit), and unstable periodic MMOs 1^7^ and 1^8^ (gray orbits), together with ribbons $R_{6}$, $R_{7}$ and $R_{8}$ (colored surfaces), respectively. The insets show enlargements near the line $L_{2}^{a}$ where the twin canard orbits $\xi _{i}$ and $\xi '_{i}$ (thick curves) are labeled. Starting from the lower part of a given periodic MMO $1^{i}$ in all panels, the MMO stays very close to the ribbon $R_{i}$ in between the bounding twin canard orbits $\xi _{i}$ and $\xi '_{i}$ and makes *i* SAOs before making a large excursion back to its starting point. This mechanism of MMOs was also found for the autocatalator system in [38].

### Continuation of Canard Orbits

**Fig. 20:**
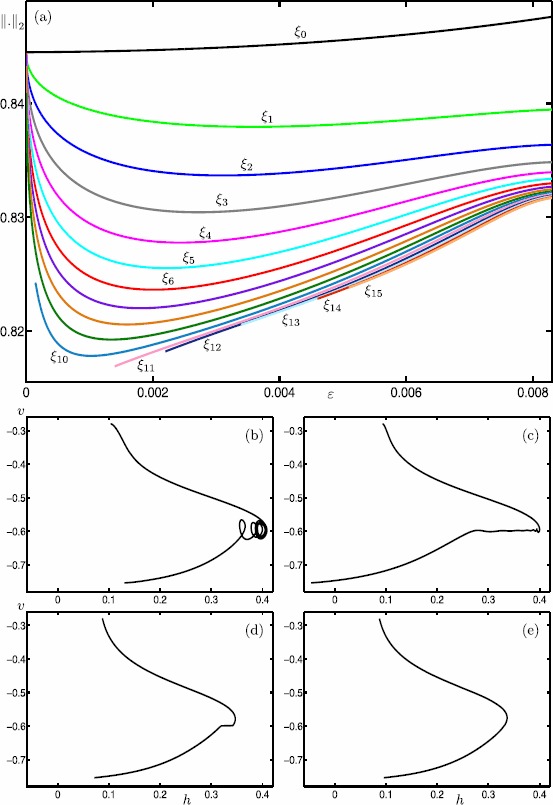
Continuation in *ε* of canard orbits $\xi _{0}$–$\xi _{15}$ in system (32) with $I=9.74$. Panel **(a)** shows the $L_{2}$-norm of the canard orbit versus *ε*. Panels **(b)**–**(e)** show the canard orbit $\xi _{6}$ for $\varepsilon =0.0083$, $\varepsilon =0.003$, $\varepsilon =3.0 \times 10^{-5}$ and $\varepsilon =1.0 \times 10^{-8}$, respectively

We now investigate the evolution of twin canard orbits as *ε* is varied. Once a canard orbit $\xi _{i}$ has been found as a solution of the well-posed 2PBVP with boundary conditions (48), it can be continued in system parameters. Figure 20 shows the continuation of canard orbits $\xi _{0}$–$\xi _{15}$ in *ε*. We start from the canard orbits that we find for $\varepsilon =0.0083$ and continue them in the direction of decreasing *ε*. Panel (a) shows the $L_{2}$-norm of the canard orbits versus *ε*. In slight abuse of notation, we use $\xi _{i}$ to denote the *ε*-dependent branches that correspond to canard orbits with *i* SAOs. We find that the branches of canard orbits $\xi _{0}$–$\xi _{9}$ can all be continued to the singular limit, and the respective canard orbits all converge to the strong singular canard $\xi _{s}$ in the limit as $\varepsilon \to 0$, as predicted by the theory [34]. Because of this accumulation onto $\xi _{s}$, the continuation of canard orbits with larger numbers of SAOs becomes a very delicate task numerically as *ε* decreases, which is why $\xi _{10}$–$\xi _{15}$ do not reach the singular limit $\varepsilon =0$.

Panels (b)–(e) of Fig. 20 demonstrate the evolution of the canard orbit $\xi _{6}$ in projection onto the ($h,v$)-plane for $\varepsilon =0.0083$, $\varepsilon =0.003$, $\varepsilon =3.0 \times 10^{-5}$ and $\varepsilon =1.0 \times 10^{-8}$, respectively. As the singular limit $\varepsilon =0$ is approached, the mechanism for SAOs of the canard orbits is clearly directly related to the folded node *p*, as is expected from the theory [33, 34]. More precisely, the SAOs occur near *p* and away from *q*. Also, the amplitudes of the SAOs become infinitely small as $\varepsilon \to 0$. This is another indication that our computational technique is reliable for computing canard orbits and saddle slow manifolds. The transition of canard orbit $\xi _{6}$ in panels (b)–(e) is representative of the other canard orbits $\xi _{1}$–$\xi _{15}$.

**Fig. 21:**
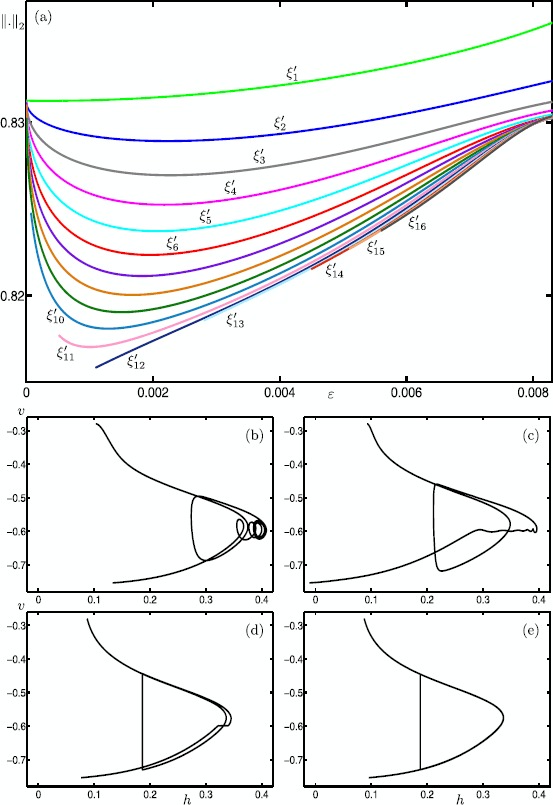
Continuation in *ε* of the twins $\xi '_{1}$–$\xi '_{16}$ of canard orbits $\xi _{1}$–$\xi _{16}$, for system (32) with $I=9.74$. Panel **(a)** shows the $L_{2}$-norm of the canard orbits versus *ε*. Panels **(b)**–**(e)** show the canard orbit $\xi '_{6}$ for $\varepsilon =0.0083$, $\varepsilon =0.003$, $\varepsilon =3.0 \times 10^{-5}$ and $\varepsilon = 2.39441 \times 10^{-8}$, respectively

Figure 21(a) shows the continuation of canard orbits $\xi '_{1}$–$\xi '_{16}$; the branches of these canard orbits all go toward $\varepsilon =0$, but the orbit segments do not converge to the strong singular canard $\xi _{s}$. This is illustrated in Fig. 21(b)–(e), where we demonstrate the evolution of the canard orbit $\xi '_{6}$ as *ε* decreases to 0. Here, $\xi '_{6}$ is shown in projection onto the ($h,v$)-plane for $\varepsilon =0.0083$, $\varepsilon =0.003$, $\varepsilon =3.0 \times 10^{-5}$ and $\varepsilon =3.52578 \times 10^{-8}$, respectively. Panel (c) shows that the last SAO of the canard orbit $\xi '_{6}$ has already become much larger than the other SAOs. In panel (d), the last oscillation of $\xi '_{6}$ becomes even larger and involves concatenations of slow and fast segments. Note that the canard orbit $\xi '_{6}$ now looks like a canard orbit $\xi _{5}$ followed by a fast return segment to a canard orbit $\xi _{0}$. The amplitudes of the first five SAOs become infinitely small in the limit as $\varepsilon \to 0$. As a result, in the limit $\varepsilon = 0$, the canard orbit $\xi '_{6}$ is a concatenation of slow and fast segments of the reduced system (34) and the fast subsystem (33), respectively. This difference in evolution, as compared to $\xi _{6}$, is representative for all twin canard orbits $\xi '_{1}$–$\xi '_{16}$, and was also found in the reduced three-dimensional Hodgkin–Huxley model; see [36].

**Fig. 22:**
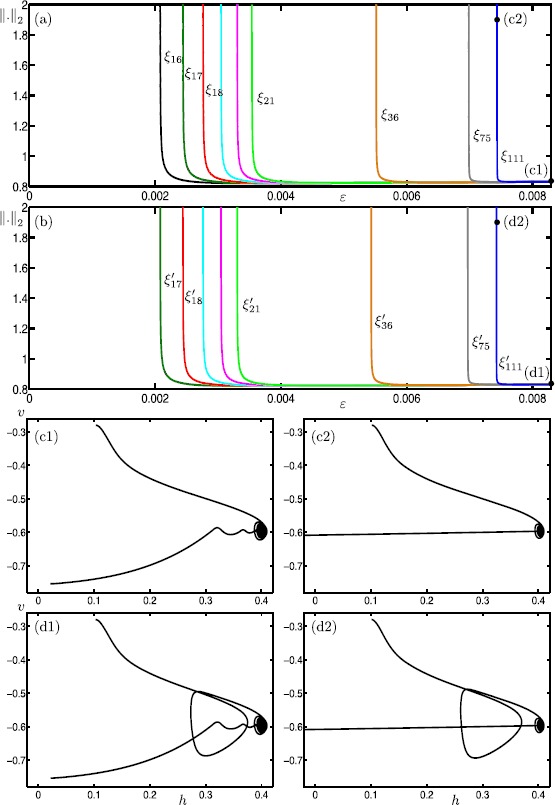
Continuation in *ε* of canard orbits **(a)** $\xi _{16}$–$\xi _{21}$, $\xi _{36}$, $\xi _{75}$ and $\xi _{111}$ and **(b)** $\xi '_{17}$–$\xi '_{21}$, $\xi '_{36}$, $\xi '_{75}$ and $\xi '_{111}$ in system (32) with $I=9.74$. Panels **(a)** and **(b)** shows the $L_{2}$-norm of the canard orbits versus *ε*. Panels **(c1)** and **(c2)** show the canard orbit $\xi _{111}$ for $\varepsilon =0.0083$ and $\varepsilon =7.43657 \times 10^{-3}$, respectively. Panels **(d1)** and **(d2)** show the canard orbit $\xi '_{111}$ for $\varepsilon =0.0083$ and $\varepsilon =7.42810 \times 10^{-3}$, respectively

Figure 22 shows the continuation of canard orbits $\xi _{16}$–$\xi _{21}$, $\xi _{36}$, $\xi _{75}$ and $\xi _{111}$, as well as $\xi '_{17}$–$\xi '_{21}$, $\xi '_{36}$, $\xi '_{75}$ and $\xi '_{111}$. Panels (a) and (b) shows the $L_{2}$-norm of the canard orbits versus *ε*. We find that the shown branches do not converge to the singular limit, as expected from the theory [34]. Instead, the $L_{2}$-norm of each branch increases asymptotically at a finite value of *ε*. This also confirms that canard orbits $\xi _{10}-\xi _{15}$ and $\xi '_{10}-\xi '_{16}$ are qualitatively different and should indeed go to the singular limit of *ε*. We remark that the limiting values of *ε*, for a given pair $\xi _{i}$ and $\xi '_{i}$ in Fig. 22(a)–(b), are close but different.

To understand this transition, panels (c1) and (d1) of Fig. 22 show the canard orbits $\xi _{111}$ and $\xi '_{111}$, respectively, in projection onto the ($h,v$)-plane for $\varepsilon =0.0083$. Notice that the mechanism for SAOs of $\xi _{111}$ and $\xi '_{111}$ is directly related to the saddle focus *q* rather than the folded node *p*. More specifically, these canard orbits approach *q* along the weak direction of the two-dimensional stable manifold $W^{s}(q)$ and make 111 SAOs around *q*, due to the rotational nature of the two-dimensional unstable manifold $W^{u}(q)$. The difference is that $\xi '_{111}$ has one final larger oscillation after the passage near *q*; compare (c1) and (d1). As *ε* decreases during the continuation, both canard orbits approach $W^{s}(q)$ much more closely; see Fig. 22(c2) and (d2). As a result, the respective starting points of the canard orbits lie on $L^{a}$ at $h \approx -21.897$, that is, far to the left, outside the range of the panels. As *ε* is decreased further, the twin canard orbits $\xi _{111}$ and $\xi '_{111}$ converge to $W^{s}(q)$ and connect to *q* along the weak stable eigendirection in the limit. Nevertheless, past *q*, the canard orbit $\xi '_{111}$ still features the final larger oscillation.

When the twin canard orbits $\xi _{i}$ and $\xi '_{i}$ are continued for increasing *ε*, we find that the respective branches have folds but do not merge with one another. This is in contrast to the autocatalator system [38], where twin canard orbits are created at fold bifurcations. Further investigation of the difference in the nature of twin canard orbits is beyond the scope of this paper and left for future research.

## Conclusions and Directions for Future Work

We introduced a general method for computing two-dimensional saddle slow manifolds and associated canard orbits in systems with two fast and two slow variables. Our approach is based on the idea of choosing boundary conditions that define submanifolds transverse to stable and unstable normal directions of the saddle slow manifold. Indeed, its novelty is to define and solve two-point boundary-value problems (2PBVPs) with boundary conditions corresponding to codimension-*i* and codimension-*j* hypersurfaces for any *i* and *j*, such that $i+j=4$. We employed and tested the resulting computational methods for an extended normal form of a folded node. Taking into account the nature of the slow flow and its normal direction, we were able to compute different relevant parts of attracting and saddle slow manifolds in the neighborhood of a folded node. The flexibility of our method also enabled us to compute two-dimensional submanifolds of the three-dimensional stable and unstable manifolds of a saddle slow manifold.

Furthermore, we illustrated how the interaction between two-dimensional slow manifolds in $\mathbb{R}^{4}$ is very similar to that found in $\mathbb{R}^{3}$; however, their intersections are no longer structurally stable. This means that canard orbits can no longer be described as intersections of two-dimensional slow manifolds. Rather, canard orbits exist as trajectories that follow attracting and saddle slow manifolds for $\mathcal{O}(1)$ time on the slow time scale before exiting via the unstable manifolds of the saddle slow manifold. We introduced a computational approach for detecting canard orbits in $\mathbb{R}^{4}$ by defining suitable 2PBVPs and employing a homotopy step to find a first solution. With this approach, we systematically detected canard orbits and continued them in the time-scale parameter *ε*, as well as the eigenvalue ratio *μ* of the folded node. Our findings demonstrate that the canard orbits of the extended normal form in $\mathbb{R}^{4}$ behave according to what the theory predicts for the three-dimensional normal form.

We demonstrated that our computational approach is also practical for studying complex slow–fast dynamics that inherently appear in applications and especially in neuroscience. Specifically, we investigated the dynamics of the full four-dimensional Hodgkin–Huxley model without applying any reduction. The bifurcation diagram of this system features various signatures of MMOs, which are organized by slow manifolds and associated canard orbits. With our approach, we were also able to compute relevant submanifolds of the attracting and saddle slow manifolds, as well as associated canard orbits in the four-dimensional phase space of the system. We showed that, for *ε* sufficiently large, the extended attracting slow manifold is subdivided into ribbons that are bounded by twin canard orbits in a similar structure to that found for the three-dimensional autocatalator model [38]. Continuation analysis of canard orbits in *ε* showed again that the relevant subset of these canard orbits converges to the strong singular canard as the singular limit is approached.

When continuing canard orbits in *ε* in the four-dimensional Hodgkin–Huxley model, we found that the twin canard orbit $\xi '_{i+1}$, for $0 \leq i < 16$, has the same limit as the canard orbit $\xi _{i}$, but with an additional large oscillation. When $\xi '_{i+1}$ can be continued all the way to the singular limit $\varepsilon =0$, this large oscillation converges to the primary strong canard $\xi _{s}$ composed with a fast return segment. This convergence process has also been found for a selection of canard orbits in the three-dimensional Hodgkin–Huxley model [36]. On the other hand, canard orbits $\xi _{i}$ and $\xi '_{i+1}$, for $i \geq 16$, do not converge to the singular limit in the four-dimensional Hodgkin–Huxley model, but connect to the saddle focus of the system as *ε* is decreased.

Overall, the work presented in this paper shows that it is possible to provide comprehensive insight into the organization of MMOs by examining the geometry of two-dimensional slow manifolds, even when the number of fast variables exceeds one. The basis of this achievement lies in a versatile construction of suitable selections of 2PBVPs, adapted to the respective context. In this way, the organization of the phase space by slow manifolds of four-dimensional slow–fast systems can be studied efficiently and comprehensively

Interactions between slow and invariant manifolds are known to play an important role in the organization of MMOs. In particular, intersections of repelling slow manifolds and unstable manifolds of saddle-focus equilibria may organize the large excursions of MMOs [72–74]. Such connecting orbits can be found with a homotopy technique similar to that used for detecting canard orbits in this paper. Understanding the nature of such intersections between different types of manifolds in the Hodgkin–Huxley model and other slow–fast systems in $\mathbb{R}^{4}$ is an interesting direction for future work.

Many examples in applications involve more than two time scales [39, 44, 75]. Furthermore, many slow–fast systems have no explicit time-scale separation and may contain various regions with different splittings of time scales [4, 5, 44, 76, 77]. In such systems, it will be necessary to study different regions locally using a variety of 2PBVP setups. These regions then need to be connected globally in order to understand the recurrent dynamics. The computational methods presented in this paper may be of use also for the investigation of such more general systems.

## Appendix

The steady-state functions of system (32) are defined by

$$ x_{\infty }(v)= \frac{\alpha _{x}(v)}{\alpha _{x}(v)+\beta _{x}(v)}, $$

for $x \in \{m, h, n\}$, where

$$\begin{aligned} \alpha _{m}(v) &= \frac{(k_{v} v + 40)/10}{1- \exp(-(k_{v} v+40)/10)}, \\ \beta _{m}(v) &= 4 \exp \bigl(-(k_{v} v+65)/18 \bigr), \\ \alpha _{h}(v) &= 0.07 \exp \bigl(-(k_{v} v+65)/20 \bigr), \\ \beta _{h}(v) &= \frac{1}{1+ \exp(-(k_{v} v+35)/10)}, \\ \alpha _{n}(v) &= \frac{(k_{v} v + 55)/100}{1- \exp(-(k_{v} v+55)/10)} \end{aligned}$$

and

$$ \beta _{n}(v) = 0.125 \exp \bigl(-(k_{v} v+65)/80 \bigr). $$

Similarly, time functions of system (32) are defined by

$$ t_{x}(v)=\frac{p_{x}}{\alpha _{x}(v)+\beta _{x}(v)}, $$

for $x \in \{m, h, n\}$, where $p_{m}=120$, $p_{h}=1$, $p_{n}=1$. The other parameters are given in Table 1; see Sect. 5. Note that the value of $p_{m}$ is different from that given in [42] so that it agrees with the nondimensionalization performed in [1] and corresponds to the classical parameter values of the original Hodgkin–Huxley model [8].

## Abbreviations

MMO: Mixed-mode oscillation
SAO: Small-amplitude oscillation
LAO: Large-amplitude oscillation
GSPT: Geometric singular perturbation theory
2PBVP: Two-point boundary-value problem
